# Placenta–Brain Axis Under Heat Stress: Inflammatory Pathways Linking Prenatal Thermal Exposure to Offspring Neurodevelopment

**DOI:** 10.3390/cells15171621

**Published:** 2026-09-07

**Authors:** Alina Liepinaitienė, Nikolaos S. Avramiotis, Dimitra Metallinou, Eirini Orovou, Angeliki Bolou, Maria Tzeli, Aikaterini Sousamli, Audrius Dėdelė, Antigoni Sarantaki

**Affiliations:** 1 Department of Environmental Sciences, Faculty of Natural Sciences, Vytautas Magnus University, LT-53361 Kaunas, Lithuania; audrius.dedele@vdu.lt; 2 SMK College of Applied Sciences, Kauno m. sav., 46326 Kaunas, Lithuania; 3 Department of Neurology and Stroke Center, University Hospital Basel, University of Basel, 4031 Basel, Switzerland; 4 Midwifery Department, Faculty of Health and Care Sciences, University of West Attica, Egaleo, 122 43 Athens, Greece; 5 Midwifery Department, University of Western Macedonia, 50 200 Kozani, Greece; 6 Faculty of Medicine, Kauno Kolegija Higher Education Institution, Kauno m. sav., 50468 Kaunas, Lithuania

**Keywords:** prenatal heat exposure, placenta–brain axis, neurodevelopment, inflammation, climate change

## Abstract

**Highlights:**

- Prenatal thermal exposure was associated with a spectrum of fetal and offspring neurodevelopmental outcomes, with the strongest and most consistent early-pregnancy evidence involving neural tube defects during the periconceptional and neurulation periods.
- Experimental evidence indicates that heat stress can disrupt placental barrier function, inflammatory and oxidative pathways, glucocorticoid and serotonin-related signaling, and fetal-brain developmental processes, supporting the biological plausibility of a heat-related placenta–brain framework.
- The placenta may represent a biologically plausible interface linking maternal thermal stress with fetal brain development. Still, current evidence does not establish a causal heat-induced placenta–brain axis in humans.
- Integrated prospective studies combining individual-level heat assessment, placental biomarkers, fetal-brain imaging, and longitudinal neurodevelopmental follow-up are needed to identify critical gestational windows and test placental mediation.

**Abstract:**

Climate change is increasing the frequency and intensity of extreme heat events, raising concern about prenatal thermal exposure as a potential risk factor for fetal brain development and offspring neurodevelopment. This systematic review aimed to synthesize evidence on prenatal heat exposure, placental inflammatory or stress-response pathways, and fetal or offspring neurodevelopmental outcomes, while evaluating the placenta–brain axis as a proposed mechanistic hypothesis rather than an established causal pathway. A systematic literature search was conducted in PubMed/MEDLINE, Scopus, Web of Science, Google Scholar, and Embase from database inception to 30 June 2026. Human observational studies, animal experiments, and in vitro mechanistic studies were included when they examined prenatal thermal exposure in relation to either placental function and stress-response mechanisms or fetal-brain and offspring neurodevelopmental outcomes. Ambient environmental heat, infectious fever, behavioral or exogenous heat exposure, experimental maternal hyperthermia, and direct cellular or organoid thermal stimulation were considered separately because these exposures are not biologically equivalent. Across the included studies, distinct prenatal thermal exposures—including ambient environmental heat, infectious fever, behavioral or exogenous heating, and experimentally induced hyperthermia—were associated with congenital central nervous system anomalies, including neural tube defects, as well as later outcomes such as neurodevelopmental delay, language impairment, autism spectrum disorder, cerebral palsy, and altered child-brain morphology. The strongest and most consistent early-pregnancy signal concerned neural tube defects during the periconceptional and neurulation periods, whereas evidence for later neurodevelopmental outcomes identified more heterogeneous susceptibility windows across gestation. Experimental evidence suggested alterations in placental barrier function, glucocorticoid and serotonin-related signaling, inflammatory and oxidative pathways, myelination, apoptosis, and neural-progenitor development. However, only one included experimental study jointly assessed maternal heat stress, placental alterations, and fetal-brain-related outcomes, while the human studies did not measure placental mediators or perform mediation analyses. Evidence from non-heat inflammatory studies therefore provides only indirect mechanistic context. Overall, prenatal heat exposure may contribute to neurodevelopmental vulnerability through multiple direct and indirect pathways, including a biologically plausible but unproven placenta–brain framework. Prospective studies integrating individual-level heat assessment, placental biomarkers, standardized fetal-brain imaging, and longitudinal neurodevelopmental follow-up are required to test this hypothesis.

## 1. Introduction

Climate change has intensified the frequency, duration, and severity of extreme heat events, making prenatal thermal exposure an increasingly important environmental risk factor for maternal, fetal, and child health. Pregnancy represents a period of heightened vulnerability to heat because maternal thermoregulation, cardiovascular adaptation, plasma volume expansion, and uteroplacental perfusion must support both maternal homeostasis and fetal growth. Recent epidemiological syntheses have shown that heat exposure during pregnancy is associated with adverse obstetric and neonatal outcomes, including preterm birth, stillbirth, congenital anomalies, fetal growth restriction, and other pregnancy complications [1,2]. However, beyond these perinatal endpoints, increasing attention is now being directed toward the possibility that prenatal heat exposure may also influence fetal brain development and later offspring neurodevelopment.

The developing brain is particularly sensitive to environmental disruption because neurogenesis, neuronal migration, synaptogenesis, myelination, and glial maturation occur within tightly regulated temporal windows. During these periods, even transient disturbances in oxygen delivery, inflammatory signaling, oxidative stress, endocrine regulation, or nutrient transport may have long-term consequences for brain structure and function. Emerging human evidence supports this concern. In a large birth cohort from China, Lin et al. reported that heat-wave exposure during early and late pregnancy was associated with an increased risk of neurodevelopmental delay in young children [3]. Similarly, data from the French Étude Longitudinale Française depuis l’Enfance (ELFE) birth cohort showed that prenatal and early-life exposure to high temperatures, particularly severe night-time heat during gestation, was negatively associated with language development at two years of age [4]. More recently, prenatal exposure to extreme heat has also been associated with autism spectrum disorder and cerebral palsy in large population-based studies [5,6]. Although these findings do not establish causality, they suggest that prenatal heat exposure may act during sensitive developmental windows and may contribute to a broader spectrum of neurodevelopmental vulnerability. Importantly, these epidemiological studies generally assessed environmental heat exposure and offspring outcomes without simultaneously measuring placental biological responses; therefore, they do not directly establish placental mediation.

A central biological question is how external thermal exposure might translate into fetal neurodevelopmental risk. The placenta is a biologically plausible candidate mediator, because it is not only an organ of gas, nutrient, and waste exchange but also an active endocrine, immune, and metabolic interface between the mother and fetus. Placental function is highly sensitive to maternal environmental stressors, including heat, hypoxia, infection, inflammation, and oxidative stress. Heat exposure may impair placental vascular function, alter nutrient transport, disrupt mitochondrial activity, increase oxidative stress, and activate inflammatory pathways [7,8]. These processes may compromise uteroplacental perfusion and fetal oxygenation while also changing the molecular signals that reach the fetal compartment. Accordingly, the placenta has been proposed as a potential biological interface linking prenatal thermal exposure with fetal brain development; however, this pathway remains a mechanistic hypothesis rather than an established causal axis.

Experimental studies provide preliminary and incomplete mechanistic evidence relevant to this hypothesis. In a mouse model, Guo et al. showed that heat stress during late pregnancy affected placental barrier function and was associated with alterations in fetal brain and intestinal function [9]. This study is particularly important because it is the only included investigation that concurrently assessed maternal heat stress, placental alterations, and fetal-brain-related outcomes within the same experimental framework. In a subsequent study, the same group demonstrated that maternal heat stress modulated placental immune-response genes, suggesting that heat exposure may induce or reshape inflammatory signaling at the maternal–fetal interface [10]. Animal evidence also shows that gestational exposure to high environmental temperature can alter offspring behavior, neurodevelopmental milestones, and gut microbiome composition [11]. However, these studies differ in exposure protocols, gestational timing, biological endpoints, and the extent to which placental and neurological outcomes were jointly evaluated. Collectively, they suggest that heat stress may not act solely through maternal hyperthermia but may also involve placental, immune, metabolic, and developmental pathways. Nevertheless, the available experimental evidence remains insufficient to demonstrate a coherent heat-induced placenta–brain pathway across models.

Inflammation is a particularly important candidate mechanism within the proposed heat-related placenta–brain framework. Maternal immune activation and placental inflammation have been repeatedly implicated in altered fetal brain development and later neurodevelopmental disorders. Experimental work has demonstrated that maternal inflammation can disrupt fetal neurodevelopment through increased placental serotonin output to the fetal brain [12]. Similarly, interleukin-1 signaling has been shown to mediate placental damage and neurodevelopmental abnormalities in offspring, while IL-1 receptor antagonism can attenuate these effects in animal models [13]. These studies did not investigate heat exposure and therefore should not be interpreted as direct evidence of heat-induced placental mediation. Rather, they provide indirect mechanistic context by identifying placental inflammatory pathways through which maternal physiological stress could plausibly influence the developing fetal brain. Heat stress is known to interact with inflammatory, oxidative, and vascular pathways, but whether it activates these specific placenta-to-brain mechanisms in human pregnancy has not yet been demonstrated.

Despite this growing evidence, the literature remains fragmented. Human epidemiological studies increasingly link prenatal heat exposure with neurodevelopmental outcomes, but most do not include placental biomarkers, inflammatory mediators, or fetal neuroimaging measures [3,4,5,6,8]. Conversely, animal and mechanistic studies provide evidence that heat stress can alter placental function, immune signaling, and fetal developmental pathways, but they often do not assess long-term neurodevelopmental phenotypes [7,8,9,10,11]. Moreover, the available literature encompasses heterogeneous forms of thermal exposure, including ambient environmental heat, heat waves, infectious fever, sauna or hot-tub exposure, experimentally induced maternal hyperthermia, and direct thermal stimulation in cellular or organoid models. These exposures are not biologically equivalent and require separate consideration, particularly because fever is strongly confounded by infection and systemic inflammation. As a result, the proposed placenta–brain pathway under heat stress remains supported primarily by partially overlapping and indirect lines of evidence rather than by direct, integrated human data. A systematic synthesis of human, animal, and experimental evidence is therefore needed to evaluate the extent to which these separate evidence streams support a plausible mechanistic framework, while clearly distinguishing direct evidence from indirect biological inference.

The aim of this review is to synthesize the available evidence on the relationship between prenatal heat or thermal stress exposure, placental inflammatory and stress-response mechanisms, and fetal or offspring neurodevelopmental outcomes. The review does not assume that a heat-induced placenta–brain axis has been established. Instead, it evaluates the placenta–brain axis as a proposed mechanistic hypothesis by integrating three distinct evidence domains: studies of prenatal heat exposure and offspring neurodevelopment, studies of heat-related placental dysfunction, and indirect mechanistic studies of placental signaling and fetal brain development. The review also identifies critical exposure windows, candidate biological pathways, major methodological limitations, and priorities for integrated prospective research.

## 2. Materials and Methods

### 2.1. Study Design and Protocol Registration

This systematic review was designed and reported in accordance with the Preferred Reporting Items for Systematic Reviews and Meta-Analyses statement, PRISMA 2020 [14]. The review protocol was prospectively registered in the International Prospective Register of Systematic Reviews (PROSPERO) under registration number CRD420261435152 on 27 June 2026. The review was planned as a systematic review with narrative and mechanistic synthesis because substantial heterogeneity was expected across human epidemiological studies, animal experiments, and mechanistic placental or neurodevelopmental models.

Any deviations from the registered protocol are documented and justified in Supplementary Table S1. These included any changes to the eligibility criteria, exposure categorization, outcome grouping, risk-of-bias assessment, and synthesis approach. Any post-registration decision to distinguish ambient heat, fever-related hyperthermia, behavioral or exogenous heat exposure, experimental maternal hyperthermia, and direct cellular or organoid thermal stimulation was reported transparently.

### 2.2. Search Strategy

A systematic literature search was performed in PubMed/MEDLINE, Scopus, Web of Science, Google Scholar, and Embase from database inception to 30 June 2026. Additional records were identified through manual screening of the reference lists of eligible articles and relevant reviews. The search strategy was designed to capture studies examining prenatal heat or thermal exposure in relation to either placental inflammatory or stress-response pathways or fetal-brain and offspring neurodevelopmental outcomes.

The search algorithm was structured around four main concept groups. The first concept group included terms related to pregnancy and prenatal exposure, such as pregnancy, pregnant, prenatal, antenatal, gestational, maternal exposure, and in utero exposure. The second concept group included terms related to heat and thermal exposure, including heat stress, heatwave, heat wave, extreme heat, ambient temperature, high temperature, hyperthermia, and thermal stress. The third concept group included terms related to placental biology and mechanistic pathways, including placenta, placental function, placental barrier, maternal–fetal interface, inflammation, cytokines, immune activation, oxidative stress, heat-shock proteins, and stress-response pathways. The fourth concept group included terms related to fetal brain development and offspring neurodevelopment, including fetal brain, brain development, brain morphology, neurodevelopment, neurodevelopmental delay, child development, cognition, language development, behaviour/behavior, autism spectrum disorder, cerebral palsy, and other neurodevelopmental outcomes.

To ensure consistency with the eligibility criteria and maximize search sensitivity, two complementary search streams were used rather than requiring all four concept groups to be present simultaneously. The first stream combined pregnancy- or prenatal-exposure terms AND heat- or temperature-related terms AND placental or mechanistic terms. The second stream combined pregnancy- or prenatal-exposure terms AND heat- or temperature-related terms AND fetal-brain or neurodevelopmental terms. Within each concept group, synonyms and related terms were combined using “OR”.

The search syntax was adapted for each database according to its indexing system, controlled vocabulary, platform, and field requirements. The complete reproducible search strategy for each database, including the exact search strings, controlled-vocabulary terms, free-text terms, search fields, database platform, date of execution, and number of records retrieved, is provided in Supplementary Table S2.

Google Scholar was searched using predefined combinations of terms related to pregnancy, prenatal heat exposure, placental mechanisms, fetal brain development, and offspring neurodevelopment. The exact Google Scholar queries, search date, result-ordering method, and number of records screened are reported in Supplementary Table S2. For each query, the first 200 results, ranked by relevance, were screened. This limit was prespecified because Google Scholar provides a large and dynamically ranked result set and does not offer the same reproducibility, indexing structure, or advanced search-field control as conventional bibliographic databases.

No restriction was applied to the study design at the search stage to maximize sensitivity. Records were subsequently screened according to the predefined eligibility criteria.

### 2.3. Eligibility Criteria

Studies were considered eligible if they were original research articles evaluating prenatal, antenatal, or gestational exposure to heat, high ambient temperature, heat waves, hyperthermia, or experimentally induced thermal stress in relation to either placental function or inflammatory/stress-response mechanisms, or fetal brain development or offspring neurodevelopmental outcomes.

Eligible study designs included human observational studies, such as cohort, case-control, nested case-control, cross-sectional, and ecological studies, as well as animal studies and experimental mechanistic models, including placental, fetal-brain, or brain-organoid models. Human studies were eligible if the exposure occurred during pregnancy or if prenatal exposure could be separated from postnatal exposure. Animal studies were eligible if heat or thermal stress was applied during gestation and outcomes were assessed in the placenta, fetus, or offspring. Mechanistic experimental studies were eligible if they provided biologically relevant evidence on heat-induced placental dysfunction, inflammatory activation, oxidative stress, immune signaling, or fetal neurodevelopmental disruption.

The eligible exposures were classified a priori into distinct categories: (1) ambient environmental heat or heat-wave exposure; (2) maternal infectious fever or febrile illness; (3) behavioral or exogenous heat exposure, including sauna, hot-tub, electric-blanket, or similar sources; (4) experimentally induced maternal hyperthermia or gestational heat stress in animal models; and (5) direct thermal stimulation in cellular, placental, fetal-brain, or organoid models. These exposure categories were not treated as biologically interchangeable. In particular, fever-related hyperthermia was considered separately because infection and systemic inflammation may independently affect placental and fetal-brain outcomes.

The main mechanistic outcomes included placental inflammation, cytokine expression, immune activation, oxidative stress, heat-shock protein expression, mitochondrial dysfunction, placental vascular dysfunction, impaired placental barrier function, altered nutrient or oxygen transport, and endocrine or serotonin-related placental pathways.

Neurodevelopmental outcomes were grouped into three distinct domains: (1) congenital central nervous system anomalies, including neural tube defects; (2) fetal-brain molecular, biometric, structural, or morphological outcomes; and (3) postnatal neurodevelopmental or neurological outcomes, including neurodevelopmental delay, cognition, behavior, language development, autism spectrum disorder, cerebral palsy, schizophrenia-spectrum outcomes, and postnatal brain morphology. Neural tube defects were analyzed separately from later neurodevelopmental outcomes because they represent early embryological malformations rather than postnatal neurodevelopmental phenotypes.

Studies were excluded if they were reviews, editorials, commentaries, letters without original data, conference abstracts without sufficient methodological information, non-pregnancy studies, studies assessing heat exposure only after birth without separable prenatal exposure, studies without placental, fetal, or offspring developmental outcomes, and studies not available in English. Studies focused exclusively on maternal heat illness without fetal, placental, or offspring outcomes were also excluded.

Studies that did not investigate heat or thermal exposure but addressed potentially relevant placenta-to-brain inflammatory mechanisms were not considered part of the primary heat-exposure evidence base. Where discussed, such studies were clearly identified as indirect, non-heat mechanistic evidence and were used only to contextualize biological plausibility.

### 2.4. Study Selection

All records identified through bibliographic database searches and other identification methods were imported into reference-management software, and duplicates were removed before screening. A total of 1248 records were identified through database searches in PubMed/MEDLINE, Scopus, Web of Science, Google Scholar, and Embase. An additional 26 records were identified through manual screening of the reference lists of eligible articles and relevant reviews. After removal of 312 duplicates, 962 records remained for title and abstract screening.

Records identified through bibliographic databases and those identified through other methods were tracked separately in accordance with the PRISMA 2020 flow-diagram structure.

Titles and abstracts were screened independently by two reviewers according to the predefined eligibility criteria. During this stage, 891 records were excluded because they were not relevant to prenatal heat or thermal exposure, did not assess eligible placental, fetal-brain, or offspring neurodevelopmental outcomes, were review articles, editorials, conference abstracts, commentaries, or were otherwise outside the scope of the review.

A total of 71 reports were sought for retrieval. Three reports could not be retrieved and were classified as “reports not retrieved” rather than as full-text exclusions. Consequently, 68 full-text reports were assessed for eligibility.

After full-text assessment, 54 reports were excluded. Reasons for exclusion included the absence of eligible prenatal heat or thermal exposure; the absence of a relevant placental, fetal-brain, or offspring neurodevelopmental outcome; an ineligible study design or experimental model; duplicate or overlapping study populations; or insufficient information for eligibility assessment or data extraction. Finally, 14 studies met the inclusion criteria and were included in the qualitative synthesis. These comprised human observational studies, animal experimental studies, and in vitro mechanistic studies relevant to prenatal thermal exposure, placental stress or inflammatory pathways, and offspring neurodevelopment.

**Figure 1:**
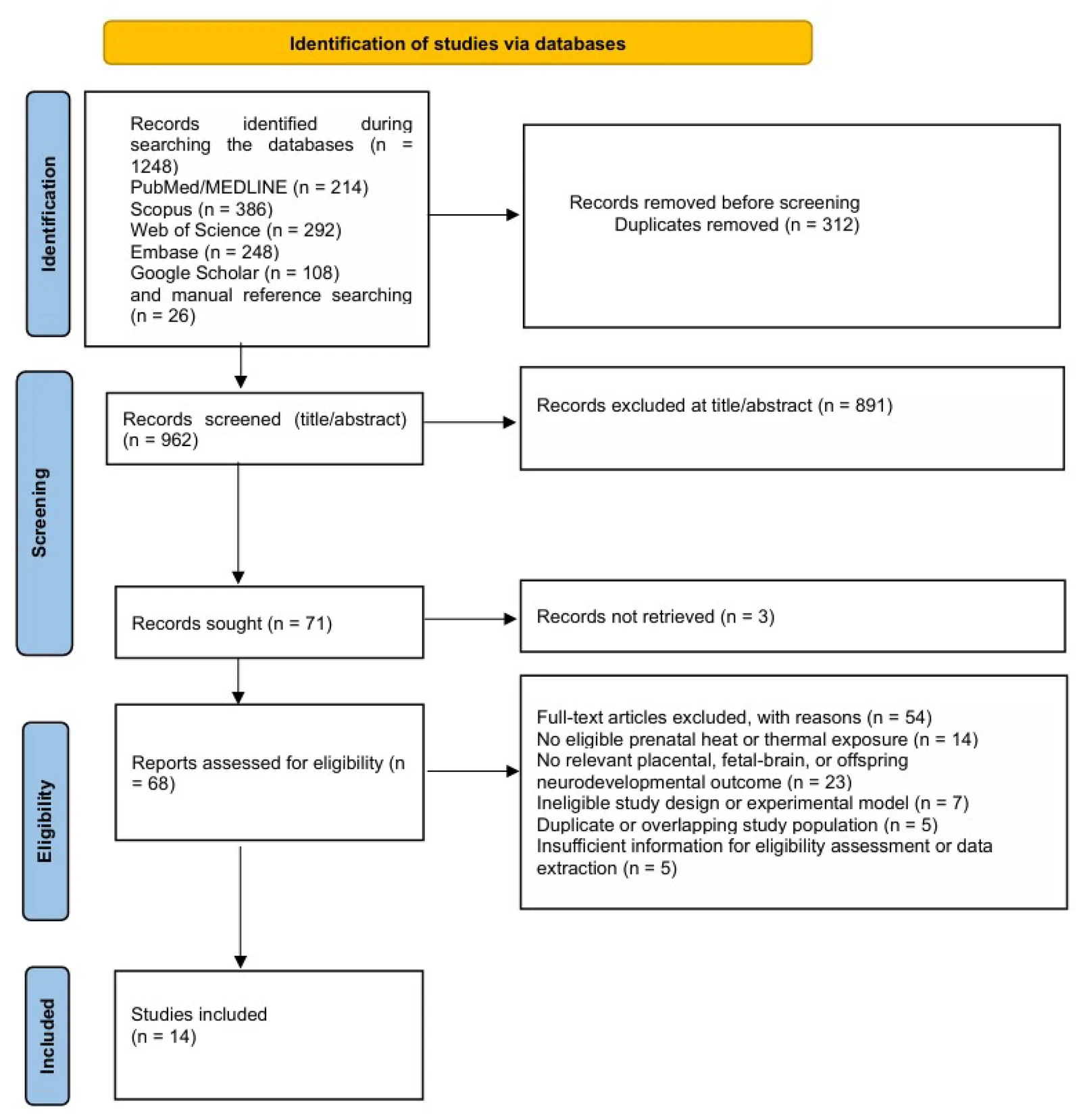
PRISMA 2020 flow diagram. Of the identified records, 1248 were retrieved through biblio-graphic database searches, and 26 additional records were identified through manual citation and reference-list searching; these identification routes were tracked separately during study selection.

Any disagreement between reviewers during title and abstract screening or full-text assessment was resolved through discussion and, when necessary, consultation with a third reviewer. The study-selection process was documented using a PRISMA 2020 flow diagram (Figure 1), including the numbers of records identified, screened, and excluded; reports sought for retrieval and not retrieved; full-text reports assessed and excluded; and studies finally included in the synthesis. Reasons for full-text exclusion were recorded.

### 2.5. Quality Assessment and Risk of Bias

The methodological quality and risk of bias of the included studies were assessed according to study design. Because the review included heterogeneous evidence from human observational studies, animal experiments, and in vitro mechanistic models, different appraisal tools were applied to each evidence category. Human observational studies were assessed using the Risk Of Bias In Non-randomized Studies of Exposures (ROBINS-E) tool (The ROBINS-I tool) [15]. In accordance with ROBINS-E terminology, domain-level and overall judgments were classified as low risk of bias, some concerns, high risk of bias, or very high risk of bias. The seven ROBINS-E domains concerned bias due to confounding, measurement of the exposure, selection of participants into the study or analysis, post-exposure interventions, missing data, measurement of the outcome, and selection of the reported result. Because ROBINS-E assessments are result-specific, judgments were based on the principal exposure–outcome result relevant to this review.

Animal experimental studies were assessed separately using the SYRCLE risk-of-bias tool [16], and the human brain-organoid study was assessed using a modified OHAT approach [17] supplemented by ToxRTool criteria [18,19]. Because these tools use different appraisal frameworks, their judgments were not interpreted as directly interchangeable with the ROBINS-E categories. The criteria used for each evidence type and the study-level judgments are reported separately in Supplementary Table S3.

Two reviewers independently completed the assessments. Disagreements were resolved through discussion and consensus, with consultation of a third reviewer when necessary. Risk of bias was not used as an eligibility threshold, and inclusion in the review did not imply that a study was free from serious methodological limitations. The reassessment produced variable overall judgments rather than a uniform moderate classification. Three human studies were judged to have serious overall risk of bias, whereas the remaining studies were judged to have moderate overall risk of bias. The complete domain-level assessment is provided in Supplementary Table S3.

### 2.6. Data Extraction

Data were extracted using a standardized data-extraction form developed specifically for this review. For each eligible study, the following information was collected: first author, year of publication, country, study design, study population or experimental model, sample size, exposure definition, exposure-assessment method, timing or gestational window of exposure, comparator or reference group, placental outcomes, inflammatory or stress-response biomarkers, fetal or offspring neurodevelopmental outcomes, follow-up duration, statistical methods, confounders or covariates, main findings, and limitations.

For human studies, additional extracted variables included maternal age, parity, socioeconomic indicators, seasonality, air-pollution adjustment, temperature metric, lag structure, trimester-specific exposure, outcome-assessment tool, child age at assessment, and reported effect estimates with confidence intervals. The exposure source was also classified as ambient heat, infectious fever, sauna or hot-tub exposure, other behavioral heating, or another defined thermal exposure.

For animal studies, extracted variables included species, strain, gestational timing of heat exposure, temperature and duration of exposure, maternal physiological responses, fetal or offspring sex when reported, placental molecular findings, fetal-brain findings, behavioral or neurodevelopmental testing, and housing or environmental conditions. For mechanistic studies, extracted data included the experimental model, thermal-stimulation protocol, molecular pathways assessed, and relevance to placental or neural development.

The extraction form also recorded whether each study assessed: (1) heat exposure and placental outcomes; (2) heat exposure and fetal-brain or offspring outcomes; or (3) heat exposure, placental responses, and fetal-brain outcomes within the same study. This distinction was used to identify direct integrated evidence separately from parallel or indirect evidence streams.

When multiple publications reported overlapping populations or datasets, the most comprehensive or most recent report was prioritized, while complementary information from related publications was extracted when relevant.

Data extraction was performed independently by two reviewers using the standardized extraction form. Discrepancies were resolved through discussion and consensus, with consultation of a third reviewer when agreement could not be reached.

### 2.7. Data Synthesis and Analysis

Because the included evidence was expected to be heterogeneous with respect to study design, exposure definition, biological model, outcome measurement, and timing of assessment, the primary synthesis was narrative and mechanistic rather than quantitative.

The synthesis was structured first by exposure category and then by outcome domain. Exposure categories comprised ambient environmental heat or heat waves, infectious fever, behavioral or exogenous heating, experimental maternal hyperthermia, and direct cellular or organoid thermal stimulation. Outcome domains comprised congenital CNS anomalies and neural tube defects, fetal-brain biometry or morphology, postnatal neurodevelopmental or neurological outcomes, placental inflammatory or stress-response outcomes, and integrated experimental evidence jointly assessing heat, placenta, and fetal-brain outcomes.

Human observational studies were synthesized separately from animal and in vitro experimental studies. Direct heat-related evidence was also distinguished from indirect, non-heat mechanistic evidence.

Within each group, findings were summarized according to study design, exposure window, biological pathway, outcome domain, and direction of association. Particular attention was given to sensitive gestational windows, including early pregnancy, placentation, mid-gestation neurodevelopment, and late-pregnancy fetal-brain maturation. Where available, sex-specific findings, dose-response relationships, and trimester-specific associations were extracted and discussed.

A formal meta-analysis was not planned because preliminary assessment indicated substantial clinical and methodological heterogeneity across studies. Differences were expected in heat-exposure metrics, such as daily maximum temperature, minimum temperature, heat-wave definitions, apparent temperature, fever, sauna or hot-tub exposure, and experimentally induced heat stress; in outcome definitions, including congenital anomalies, developmental screening tools, clinical diagnoses, neuroimaging markers, and molecular endpoints; and in study populations or experimental models. Therefore, statistical pooling would risk producing misleading summary estimates. If, during data extraction, a sufficiently homogeneous subset of human studies was identified, a random-effects meta-analysis would be considered as a secondary exploratory analysis.

The narrative synthesis focused on consistency, direction, and biological coherence of findings rather than on statistical pooling alone. The proposed placenta–brain axis was evaluated as a mechanistic framework rather than as an established causal pathway. Evidence was mapped across three distinct domains: heat exposure and offspring neurodevelopment, heat exposure and placental dysfunction, and placental signaling in relation to fetal brain development.

The strength of inference depended on whether these domains were evaluated concurrently within the same study or represented separate, indirectly connected evidence streams. Particular emphasis was placed on the fact that only one included study jointly assessed maternal heat stress, placental alterations, and fetal-brain-related outcomes, while the human epidemiological studies did not measure placental mediators or conduct formal mediation analyses.

The strength of evidence was interpreted in relation to study quality, risk of bias, reproducibility across models, exposure timing, biological relevance, and concordance between human and experimental findings.

## 3. Results of the Systematic Review

### 3.1. Overview of Included Evidence

The included studies [3,4,5,6,9,11,20,21,22,23,24,25,26,27] examined prenatal heat or thermal exposure in relation to fetal brain development, neurodevelopmental outcomes, congenital central nervous system (CNS) anomalies, placental function, and mechanistic biological pathways. The evidence included human observational studies, experimental animal studies, and an in vitro human brain-organoid model. Human studies evaluated outcomes ranging from neural tube defects (NTDs) to neurodevelopmental delay, language development, autism spectrum disorder (ASD), cerebral palsy (CP), and child brain morphology. Because NTDs represent early embryological malformations rather than postnatal neurodevelopmental phenotypes, they were synthesized separately from fetal-brain morphology and later neurodevelopmental outcomes. Experimental studies provided mechanistic evidence on placental barrier disruption, inflammatory and oxidative-stress responses, altered fetal-brain stress signaling, impaired myelination, delayed neurodevelopmental milestones, and abnormal neural-progenitor development.

The included studies also differed substantially in the type of thermal exposure assessed. Human studies examined ambient environmental heat and heat waves, infectious fever, and behavioral or exogenous heat exposure, including hot-tub, sauna, and electric-blanket use. Animal studies evaluated experimentally induced gestational heat stress, whereas the organoid study investigated direct thermal stimulation. These exposure categories were analyzed separately because they are not biologically equivalent, and fever is additionally confounded by infection and systemic inflammation.

Overall, the findings suggest that prenatal heat exposure may affect neurodevelopment through both direct and indirect pathways. The strongest human evidence was observed for early gestational exposure, particularly during the periconceptional period and the first weeks of pregnancy, which overlap with neural tube closure and early brain patterning. Later gestational windows were also implicated, especially for language development, ASD, and broader neurodevelopmental delay. However, the evidence did not establish a causal heat-induced placenta–brain axis in humans. No included human study measured placental mediators or performed formal mediation analysis, and only one included experimental study jointly assessed maternal heat stress, placental alterations, and fetal-brain-related outcomes [9].

Mechanistic studies provided partial biological support for a proposed heat-related placenta–brain framework by showing that maternal heat stress may alter placental barrier function, glucocorticoid and serotonin-related signaling, inflammatory and oxidative pathways, and fetal-brain developmental processes [9,11,26]. Nevertheless, these findings arose from different models and evidence streams. The animal study by Guo et al. [9] provided the only integrated heat–placenta–fetal-brain evidence, whereas Adebiyi et al. [11] primarily assessed offspring neurodevelopmental, inflammatory, oxidative, and myelination-related outcomes, and Xu et al. [26] examined direct thermal effects in brain organoids without maternal or placental components. Therefore, the mechanistic evidence should be interpreted as supportive of biological plausibility rather than as proof of a unified causal pathway.

The main characteristics of the included studies are summarized in Table 1, whereas the complete study-level characteristics are provided in Supplementary Table S4.

### 3.2. Prenatal Heat Exposure and Neural Tube Defects

Several human studies evaluated distinct forms of maternal thermal exposure, including infectious fever, hot-tub or sauna use, and ambient environmental heat, in relation to NTDs. Because these exposures differ substantially in source, biological mechanism, and susceptibility to confounding, their findings are presented separately rather than interpreted as evidence from a single homogeneous exposure category.

The earliest evidence came from Shiota, who examined human embryos with NTDs and reported a higher frequency of maternal febrile illness among affected embryos than among control embryos [20]. Maternal febrile illness was reported in 16 of 113 NTD cases, corresponding to 14.2%. The association appeared strongest for exencephaly with or without myeloschisis, for which febrile illness was reported in 18.0% of cases. In most cases with known fever timing, maternal illness occurred during the critical period of neural tube closure, between 17 and 43 days after conception [20]. Although this study was limited by the absence of precise maternal-temperature measurements and multivariable adjustment, it provided early human evidence that febrile illness accompanied by maternal hyperthermia during early embryogenesis may be associated with NTDs. However, the independent contribution of elevated maternal temperature cannot be separated from that of the underlying infection, inflammatory response, or associated treatment.

Milunsky et al. provided prospective evidence in a large cohort of pregnant women [21]. Heat-related exposures during early pregnancy included hot-tub use, sauna use, electric-blanket use, and fever. Exposure to a hot tub, sauna, or fever during early pregnancy was associated with an adjusted relative risk (RR) of 2.2 for NTDs. Hot-tub exposure showed the strongest individual association, with an adjusted RR of 2.8, whereas exposure to two heat sources was associated with a substantially higher risk, with an RR of 6.2 [21]. These findings support the hypothesis that maternal hyperthermia during the first trimester, particularly during the period of neural tube closure, may increase the risk of NTDs. Nevertheless, the combined exposure category incorporated both infectious fever and exogenous heating, and these estimates should not be interpreted as demonstrating equivalent effects across the different exposure sources.

Evidence from studies of ambient environmental temperature was more heterogeneous. Auger et al. examined elevated outdoor temperature during the third and fourth weeks post conception in a large retrospective cohort from Quebec [22]. Overall, maximum weekly temperature during weeks 3–4 post conception was not clearly associated with NTDs. However, day-specific analyses suggested that exposure to 30 °C versus 20 °C toward the end of the fourth week post conception was associated with increased risk, with prevalence ratios (PRs) of 1.56 and 1.49 on two consecutive days [22]. These findings indicate a possible weak association during a very narrow embryological window, although the authors emphasized the need for more precise exposure timing and temperature assessment.

In contrast, Soim et al. did not observe a significant overall association between weather-related extreme heat events (EHEs) and NTDs in a population-based case-control study from the National Birth Defects Prevention Study [23]. EHEs during the third and fourth weeks post conception were not associated with increased NTD risk, and no dose–response relationship was observed for the frequency or duration of EHEs [23]. However, exposure misclassification may have occurred because ambient heat indices do not accurately capture individual maternal exposure, indoor temperature, access to cooling, behavioral adaptation, or physiological heat responses.

More recent evidence from LaPointe et al. suggested a positive dose–response association between extreme ambient heat during the periconceptional period and NTDs [24]. In this matched case-control study from Georgia, exposure to increasing consecutive days of extreme apparent heat was associated with progressively higher odds of NTDs. Compared with no extreme heat exposure, adjusted odds ratios increased from 1.09 for 1–2 consecutive days to 1.29 for six or more consecutive days. Associations were largely driven by spina bifida, with an adjusted odds ratio of 1.41 for six or more consecutive days of exposure [24]. Weekly analyses suggested stronger associations during the early postconceptional period, particularly around gestational week 5 for spina bifida [24].

Taken together, evidence concerning congenital CNS anomalies suggests that the periconceptional and neurulation periods may represent sensitive developmental windows for thermal disruption. However, the findings remain inconsistent and vary according to whether exposure was defined as infectious fever, behavioral or exogenous heating, or ambient environmental temperature. Because NTDs are early embryological malformations, these findings were not combined conceptually with later fetal-brain morphology or postnatal neurodevelopmental outcomes.

### 3.3. Prenatal Heat Exposure and Neurodevelopmental Delay

Large human cohort studies increasingly suggest that prenatal heat exposure may be associated with later neurodevelopmental delay. Lin et al. evaluated 67,453 mother–child pairs in China and reported that heat-wave exposure during pregnancy was associated with an increased risk of neurodevelopmental delay in young children [3]. The associations were most evident for heat-wave exposure during the first and third trimesters. First-trimester exposure was associated with increased odds of neurodevelopmental delay across most heat-wave definitions, with odds ratios (ORs) ranging from 1.186 to 1.383 [3]. Third-trimester associations were also observed but were generally smaller, whereas second-trimester findings were less consistent.

Importantly, the results remained broadly stable after adjustment for particulate matter with an aerodynamic diameter ≤2.5 μm (PM2.5), nitrogen dioxide (NO2), and postnatal heat-wave exposure, suggesting that the observed associations were not explained solely by postnatal heat exposure or the measured environmental confounders [3]. However, the study did not assess placental biomarkers, inflammatory mediators, or fetal-brain imaging, and therefore provides epidemiological evidence of an association rather than direct evidence of placental mediation.

Cai et al. also reported an association between prenatal and postnatal heat exposure and suspected developmental delay (SDD) in a nationwide Chinese preschool cohort [27]. Prenatal exposure to moderate and extreme heat across the entire pregnancy was associated with higher odds of SDD. Compared with the minimum-risk temperature, prenatal moderate heat was associated with an OR of 1.28, whereas prenatal extreme heat was associated with an OR of 1.35 [27]. Associations were observed across multiple developmental domains, including communication, gross motor, fine motor, problem-solving, and personal–social development.

Although postnatal heat exposure showed stronger associations, prenatal heat remained associated with SDD after adjustment for a wide range of child, maternal, socioeconomic, environmental, and air-pollution covariates [27]. Because this study used retrospective exposure assignment and parent-completed screening questionnaires, the findings should be interpreted cautiously. In addition, suspected developmental delay identified through a screening instrument should not be considered equivalent to a confirmed clinical neurodevelopmental diagnosis.

Taken together, these studies suggest that prenatal ambient heat exposure may be associated with broad developmental vulnerability, particularly after exposure during early or late gestation. Nevertheless, the evidence remains observational, outcome definitions differ between studies, and neither study directly evaluated the placenta as a mediator between maternal heat exposure and child neurodevelopment.

### 3.4. Prenatal and Early-Life Heat Exposure and Language Development

Barbalat et al. investigated temperature exposure in the French Longitudinal Study of Children, the ELFE birth cohort, and assessed language development at two years of age using the MacArthur–Bates Communicative Development Inventories (MB-CDI) [4]. The most relevant prenatal finding was that severe night-time heat during gestational weeks 14–19 was associated with lower vocabulary production scores at age two. Severe night-time heat was associated with an RR of 0.968 for vocabulary score, corresponding to an estimated 3.2% decrease [4]. Similar negative associations were observed for moderate and extreme night-time heat, whereas daytime and overall prenatal heat exposure were not clearly associated with poorer language outcomes.

These findings suggest that night-time heat and second-trimester exposure may be particularly relevant to early language development [4]. However, postnatal heat exposure showed stronger negative associations, indicating that both prenatal and early postnatal periods may contribute to language-related neurodevelopmental outcomes. Because the study assessed both prenatal and postnatal temperature exposure, the prenatal association should be interpreted separately from the stronger postnatal findings.

The study did not measure placental biomarkers, inflammatory mediators, fetal-brain structure, or other biological intermediates. Therefore, it provides observational evidence of an association between gestational night-time heat and later language development but does not directly support placental mediation or establish a heat-induced placenta–brain pathway. In addition, the modest magnitude of the reported association and the use of parent-reported language assessment warrant cautious interpretation.

### 3.5. Prenatal Heat Exposure, Autism Spectrum Disorder, and Cerebral Palsy

Two large population-based studies examined more specific neurodevelopmental diagnoses. Luglio et al. assessed prenatal extreme heat exposure and autism spectrum disorder (ASD) in a large retrospective birth cohort from Southern California [5]. High night-time temperature, measured as weekly average minimum temperature (Tmin), was associated with an increased risk of autism diagnosis by age five. Sensitive windows were identified during both early and late pregnancy. For the 90th percentile versus the median Tmin, increased risk was observed during gestational weeks 1–7 and 32–37. For the 99th percentile, sensitive windows extended across weeks 1–10 and 30–37 [5]. Across the full pregnancy, cumulative exposure to high night-time temperature was also associated with increased autism risk. In contrast, maximum daytime temperature (Tmax) was not significantly associated with autism risk [5].

These findings suggest that night-time heat may be more relevant than daytime maximum temperature, possibly because elevated night-time temperature limits physiological recovery from daytime heat exposure. However, this explanation remains hypothetical, as the study did not directly assess maternal thermoregulation, sleep disruption, placental function, inflammatory mediators, or fetal brain development. The findings therefore support an epidemiological association between prenatal night-time heat exposure and ASD risk but do not establish a specific biological pathway.

Zhuo et al. examined high ambient temperature during pregnancy and childhood cerebral palsy (CP) in a population-based nested case-control study from California [6]. Exposure to higher weekly mean temperature during the first four gestational weeks was associated with increased CP risk. A 5 °C increase in ambient temperature during weeks 0–3 was associated with an OR of 1.08, whereas extreme heat during the same window was associated with an OR of 1.18 [6]. Cumulative trimester analyses also suggested increased risk during the first and second trimesters but not during the third trimester. Sibling analyses showed a similar early-pregnancy pattern, which strengthens the interpretation that early gestation may represent a particularly vulnerable window [6].

Nevertheless, the reported effect estimates were modest, and residual confounding, exposure misclassification, and uncertainty regarding the underlying biological mechanism remain possible. The study did not measure placental biomarkers or fetal-brain changes and therefore cannot determine whether placental dysfunction mediated the association.

Taken together, these studies suggest that prenatal ambient heat exposure may be associated with ASD and CP, with early pregnancy emerging as a potentially sensitive period and late pregnancy also appearing relevant for ASD. However, these outcomes differ substantially in pathophysiology and should not be interpreted as evidence of a single uniform neurodevelopmental effect. The available findings remain observational and do not directly establish a heat-induced placenta–brain axis.

### 3.6. Prenatal Heat Exposure and Child Brain Morphology

DeIngeniis et al. examined prenatal extreme heat exposure and child brain morphology in a small imaging pilot study from the Stress in Pregnancy (SIP) cohort [25]. Extreme heat alone was not significantly associated with basal ganglia volume. However, prenatal extreme heat appeared to moderate the association between prenatal Superstorm Sandy exposure and basal ganglia morphology. Co-exposure was associated with altered basal ganglia volumes, including reduced left nucleus accumbens volume and increased left pallidum volume [25].

Although the small sample size limits interpretation, this study is important because it provides preliminary neuroimaging evidence that prenatal heat may interact with other climate-related stressors to influence brain development. It also highlights the need to examine combined environmental exposures rather than heat in isolation. However, because the reported associations emerged in the context of co-exposure to Superstorm Sandy rather than heat exposure alone, the findings should not be interpreted as evidence of an independent effect of prenatal heat on child brain morphology. In addition, the study did not assess placental biomarkers, inflammatory mediators, or fetal brain development during gestation and therefore cannot clarify whether the observed morphological differences were mediated through placental pathways.

Overall, this study provides exploratory rather than confirmatory evidence. The small sample size, multiple imaging comparisons, and interaction-based findings substantially limit causal inference and generalizability.

### 3.7. Experimental Animal Evidence: Placental Function, Fetal-Brain Stress Response, and Offspring Neurodevelopment

Animal studies provided mechanistic support for the biological plausibility of a proposed heat-related placenta–brain framework. Guo et al. exposed pregnant Institute of Cancer Research (ICR) mice to heat stress during late gestation and found evidence of placental barrier disruption and altered fetal-brain stress signaling [9]. Heat stress increased fetal loss and altered placental nutrient transport and metabolism. It also reduced placental Hsd11b2 and Htr1d expression, suggesting disruption of the glucocorticoid barrier and serotonin-related signaling. In the fetal brain, corticosterone levels increased, and genes related to glucocorticoid and hypoxia responses were upregulated [9].

Placental and fetal tissues were assessed using RNA sequencing (RNA-seq), quantitative reverse-transcription polymerase chain reaction (qRT-PCR), Western blotting, enzyme-linked immunosorbent assay (ELISA), Gene Ontology (GO) enrichment, Kyoto Encyclopedia of Genes and Genomes (KEGG) pathway analysis, and protein–protein interaction (PPI) analyses. These findings indicate that maternal heat stress may affect the fetal brain indirectly through placental endocrine and barrier pathways. Importantly, this was the only included study that concurrently assessed maternal heat stress, placental alterations, and fetal-brain-related outcomes within the same experimental model. Accordingly, it provides the most direct evidence relevant to the proposed heat–placenta–brain pathway, although it does not establish that the placental changes causally mediated the fetal-brain effects.

Adebiyi et al. examined gestational exposure (GE) and gestational exposure plus postnatal exposure (GE + PE) to high environmental temperature in rats [11]. Heat-exposed offspring showed altered neurodevelopmental milestones and behavioral responses, including delayed ear-pinnae opening, reduced forelimb-suspension latency, reduced grasping reflex, altered hindfoot angle, and reduced body-weight gain. Heat exposure was also associated with increased inflammatory and oxidative-stress markers, including tumor necrosis factor alpha (TNF-α), interleukin-4 (IL-4), interleukin-10 (IL-10), and malondialdehyde (MDA), as well as reduced myelin basic protein (MBP) expression [11]. Additional assessed markers included neuronal nuclear antigen (NeuN), glial fibrillary acidic protein (GFAP), ionized calcium-binding adaptor molecule 1 (IBA1), and superoxide dismutase (SOD).

These findings suggest that heat exposure may influence neurodevelopment through inflammatory activation, oxidative stress, and impaired myelination. The adverse effects were more persistent in animals exposed both gestationally and postnatally, indicating that cumulative early-life heat exposure may produce greater neurodevelopmental disruption [11]. However, this study did not directly assess placental structure, placental molecular signaling, or placenta-to-brain mediation. Its findings therefore support heat-related inflammatory, oxidative, and neural-maturation mechanisms, but they should not be considered direct evidence of a placental pathway.

Together, the animal evidence supports the interpretation that prenatal heat exposure can affect neurodevelopmental processes through potentially interacting placental, endocrine, inflammatory, oxidative, and neural-maturation pathways. However, the translation of these findings to human pregnancy requires caution because of species differences, controlled exposure protocols, and variable reporting of randomization, blinding, and litter-level analytical control. The mechanistic evidence also remains fragmented: only one study jointly examined heat, placenta, and fetal-brain outcomes, whereas the remaining animal evidence primarily addressed downstream offspring or neural effects.

### 3.8. In Vitro Human Brain-Organoid Evidence

Xu et al. used human brain organoids to model periodic heat exposure during early neural development [26]. Organoids were derived from embryonic stem cells (ESCs) and induced pluripotent stem cells (iPSCs). Organoids were exposed to 40 °C twice daily during either days 10–14 (D10–14) or days 20–24 (D20–24) of development, corresponding to early neural-tube formation and later early neural-developmental stages. Periodic heat exposure reduced organoid size, impaired neural-tube-like structure development, and altered lumen volume [26].

Transcriptomic analyses showed activation of Wingless-related integration site (WNT), bone morphogenetic protein (BMP), transforming growth factor beta (TGF-β), apoptosis, and reactive oxygen species pathways, along with downregulation of cell-cycle-related signatures. Heat exposure reduced neural-progenitor proliferation markers, including SOX2, PAX6, KI67, and EdU-positive cells; increased apoptosis, as assessed by terminal deoxynucleotidyl transferase dUTP nick-end labeling (TUNEL); promoted cell-cycle exit; and increased neural-differentiation markers, including TBR1 and TBR2 [26]. Heated organoids also showed transcriptomic similarity to neurodevelopmental-disorder-related organoid models, particularly ASD-related models [26].

This study provides direct mechanistic evidence that heat exposure may disrupt early human neural development by affecting proliferation, apoptosis, differentiation, and developmental signaling pathways. Although organoid models cannot fully reproduce maternal physiology, placental function, or whole-organism neurodevelopment, they offer important human-cell-based evidence that thermal stress can directly alter early brain-developmental processes [26].

However, the organoid model did not include maternal, vascular, immune, or placental components. Therefore, the findings support a direct neural effect of thermal stress rather than a placenta-mediated mechanism. The transcriptomic similarity to ASD-related organoid models should also be interpreted cautiously, because molecular resemblance does not establish that prenatal heat exposure causes ASD or reproduces the complexity of the disorder.

### 3.9. Integrated Synthesis of the Proposed Placenta–Brain Framework Under Heat Stress

Across the included evidence, partially convergent but incomplete patterns emerge. Human observational studies suggest that prenatal heat exposure may be associated with several neurodevelopmentally relevant outcomes, including NTDs, neurodevelopmental delay, language impairment, ASD, and CP [3,4,5,6,20,21,22,23,24,27]. The most consistent sensitive window appears to be early pregnancy, particularly the periconceptional period and the first weeks of gestation, when neural-tube closure and early brain patterning occur [20,21,22,23,24]. However, later gestational windows also appear relevant, especially for language development and ASD [3,4,5].

These outcomes should not be interpreted as a single uniform neurodevelopmental phenotype. NTDs are congenital malformations arising during early embryogenesis, whereas developmental delay, language impairment, ASD, CP, and altered brain morphology represent distinct postnatal neurological or neurodevelopmental outcomes with different pathophysiological mechanisms.

Mechanistic evidence supports several possible pathways. First, maternal heat stress may impair placental barrier and endocrine function, including glucocorticoid and serotonin-related signaling [9]. Second, heat exposure may activate inflammatory and oxidative-stress pathways that may affect fetal brain development and myelination [11]. Third, direct thermal stress may alter early neural-progenitor proliferation, apoptosis, and differentiation, as shown in human brain organoids [26]. These pathways are not mutually exclusive and may interact across gestation. For example, placental dysfunction may alter fetal oxygenation, nutrient supply, and stress-hormone exposure, while inflammatory and oxidative pathways may directly affect neural maturation.

Nevertheless, the evidence does not establish that these pathways operate sequentially or causally as a unified heat-induced placenta–brain axis. Guo et al. [9] provided the only included study that jointly assessed heat stress, placental alterations, and fetal-brain-related outcomes. Adebiyi et al. [11] assessed inflammatory, oxidative, myelination, and offspring outcomes without directly testing placental mediation, whereas Xu et al. [26] evaluated direct neural effects in the absence of a placenta. Thus, the integrated framework is based largely on triangulation across separate experimental systems rather than on repeated direct demonstration of the complete pathway.

The evidence also suggests that exposure characterization is critical. Studies using self-reported fever or hot-tub exposure capture maternal hyperthermia more directly but may be limited by recall and imprecise temperature measurement [20,21]. Fever-related studies are additionally confounded by the underlying infection, maternal immune activation, inflammatory signaling, and treatment. Consequently, infectious fever should not be considered biologically equivalent to ambient environmental heat or behavioral heating.

Studies using ambient temperature or heat-wave definitions benefit from objective environmental data but may misclassify individual exposure because they do not fully capture indoor temperature, air-conditioning access, hydration, occupational exposure, maternal behavior, or physiological heat tolerance [22,23,24]. Recent studies increasingly incorporate high-resolution spatial models, trimester-specific exposure windows, air-pollution adjustment, and sensitivity analyses, strengthening the evidence base [3,4,5,6,27].

Overall, the included literature supports a biologically plausible but unproven mechanistic framework in which prenatal thermal exposure may influence fetal brain development either directly or indirectly through placental, inflammatory, endocrine, vascular, or oxidative pathways. The current evidence is insufficient to establish placental mediation in humans.

### 3.10. Risk-of-Bias Findings

Risk of bias varied across the included evidence and was not uniform across studies or study designs. Among the human observational studies assessed using ROBINS-E, Shiota et al. [20], DeIngeniis et al. [25], and Cai et al. [27] were judged to have high overall risk of bias. The principal concerns included inadequate control of confounding, imprecise or retrospective exposure assessment, very small sample size, co-exposure-related confounding, cross-sectional outcome ascertainment, and parent-reported developmental screening. The remaining human observational studies were judged as having some concerns regarding overall risk of bias, primarily because ambient-temperature models could not fully capture individual-level heat exposure and residual socioeconomic, environmental, maternal-health, or behavioral confounding remained possible.

The two animal studies, assessed using the SYRCLE risk-of-bias tool, were judged to have moderate overall risk of bias. Although they employed controlled and clearly defined heat-exposure protocols, reporting of allocation concealment, randomization, blinding, sample-size justification, and litter-level analysis was incomplete. The human brain-organoid study, evaluated using the modified OHAT/ToxRTool approach, was also judged to have moderate overall risk of bias. Its use of two pluripotent stem-cell lines, independent experiments, and multiple complementary assays supported methodological reliability; however, incomplete reporting of blinding and other methodological details, together with limited organism-level generalizability, reduced confidence. Because different appraisal tools were applied according to study design, their judgment categories should not be interpreted as directly interchangeable. Detailed domain-level assessments and tool-specific judgments are presented in Supplementary Table S3.

## 4. Discussion

This systematic review suggests that prenatal heat exposure may be associated with adverse fetal-brain and offspring neurodevelopmental outcomes. Across the included human studies, heat exposure or maternal hyperthermia was linked with neural-tube defects, neurodevelopmental delay, language impairment, autism spectrum disorder, and cerebral palsy [3,4,5,6,20,21,22,23,24,27]. Evidence for early-pregnancy vulnerability was most consistent for neural tube defects during the periconceptional and neurulation periods, while selected studies of later neurodevelopmental outcomes also identified sensitive windows in early, mid-, or late gestation [3,4,5,20,21,22,23,24]. Experimental animal and organoid studies supported biological plausibility by showing that heat stress may disrupt placental function, inflammatory and oxidative pathways, fetal stress signaling, myelination, apoptosis, and early neural development [9,11,26].

However, the evidence should not be interpreted as establishing a causal heat-induced placenta–brain axis. Only one included experimental study jointly evaluated maternal heat stress, placental alterations, and fetal-brain-related outcomes [9], and none of the included human studies measured placental mediators or performed formal mediation analyses. The proposed placenta–brain axis therefore remains a mechanistic hypothesis supported by partially overlapping epidemiological and experimental evidence.

The findings are consistent with previous evidence showing that heat exposure during pregnancy is associated with adverse maternal, fetal, and neonatal outcomes [1,2]. However, most previous syntheses have focused mainly on obstetric, fetal, or neonatal endpoints rather than later neurodevelopment [1]. Ramirez et al. specifically reviewed the impact of heat stress on placental function and supported the concept that the placenta is sensitive to thermal stress, but neurodevelopmental outcomes were not the central focus of that review [7]. Rhaman et al. recently published a scoping review on prenatal ambient heat exposure and neurodevelopment and concluded that the evidence suggests a possible association, although human studies remain limited and heterogeneous [8]. Therefore, the present review adds to the existing literature by systematically integrating human neurodevelopmental findings with placental, inflammatory, stress-response, and mechanistic evidence from animal and organoid studies [3,4,5,6,7,8,9,10,11,12,13,20,21,22,23,24,25,26,27].

This integration should nevertheless be interpreted as evidence triangulation rather than direct demonstration of one continuous biological pathway. Human epidemiological studies primarily addressed ambient heat and child outcomes, animal studies evaluated selected placental or neural mechanisms, and organoid studies tested direct cellular responses to thermal stimulation. Their convergence increases biological plausibility but does not eliminate uncertainty regarding causality, mediation, or translation to human pregnancy.

The placenta may represent a plausible, but not yet confirmed, biological interface between maternal heat exposure and fetal brain development. Heat stress may impair uteroplacental perfusion, alter placental barrier function, activate inflammatory and oxidative-stress pathways, and modify endocrine signaling [7,9,10,11]. These changes may affect fetal oxygenation, nutrient transfer, glucocorticoid exposure, and immune signaling, all of which are relevant to brain development.

Experimental evidence supports this interpretation: heat stress altered placental-barrier and fetal-brain stress-response pathways in pregnant mice [9], while gestational heat exposure in rats was associated with inflammatory activation, oxidative stress, and impaired myelination [11]. Human brain-organoid evidence further suggests that thermal stimulation can directly disturb early neural development by reducing neural-progenitor proliferation, increasing apoptosis, and altering developmental signaling pathways [26].

These studies, however, provide evidence for different components of the proposed framework. The mouse study [9] is the only included study that assessed placental and fetal-brain responses together; the rat study [11] did not directly evaluate placental mediation, and the organoid study [26] modeled a direct neural response without maternal or placental physiology.

Although maternal-inflammation studies were not heat-specific, they provide additional mechanistic context for the role of placental immune activation in fetal neurodevelopmental disruption [12,13]. They should not, however, be considered part of the direct heat-exposure evidence. These studies demonstrate that placental serotonin and interleukin-1 signaling can influence fetal brain development in inflammatory models, but they do not show that prenatal heat activates the same pathways.

The distinction among exposure types is equally important. Ambient environmental heat, infectious fever, hot-tub or sauna exposure, experimental maternal hyperthermia, and direct cellular heating differ in source, duration, intensity, physiological response, and confounding structure. Fever is particularly difficult to interpret because the underlying infection and inflammatory response may independently affect placental and fetal development. Therefore, evidence from these exposure categories should not be combined as if they represented a single homogeneous thermal exposure.

The congenital-anomaly evidence also requires separate interpretation. NTDs arise during neurulation and represent early structural malformations rather than postnatal neurodevelopmental outcomes. Their association with maternal fever or hyperthermia during the first weeks after conception is biologically and temporally distinct from later associations with language development, ASD, CP, or child-brain morphology. Reviewing these outcomes separately reduces the risk of implying a common clinical phenotype or mechanism.

These findings suggest that pregnant individuals should be considered a vulnerable population during heat waves and sustained high-temperature periods. Heat-protection advice may be particularly important before conception and during early pregnancy, when neural-tube closure and early brain development occur [20,21,22,23,24]. Reasonable precautionary measures consistent with general maternal-health guidance include avoidance of excessive exogenous heating, adequate hydration, reduced unnecessary outdoor exposure during extreme heat, and access to cooling. However, the present review did not evaluate the effectiveness of these interventions for preventing fetal or offspring neurodevelopmental outcomes [1,21,24].

Nevertheless, the preventive implications should be proportionate to the certainty of the evidence. The available data support precautionary heat-reduction measures that are already consistent with general maternal-health guidance, but they do not justify deterministic statements that prenatal heat exposure causes neurodevelopmental disorders.

The evidence also suggests that night-time heat may be important, especially for language development and autism spectrum disorder, because elevated minimum temperature may reduce maternal physiological recovery from daytime heat exposure [4,5]. This explanation remains hypothetical, as the included studies did not directly measure maternal sleep, thermoregulation, physiological recovery, placental function, or fetal stress responses.

From a research and clinical-monitoring perspective, future pregnancy studies should combine heat-exposure assessment with placental biomarkers and long-term neurodevelopmental follow-up [7,8]. Prospective studies should also integrate standardized longitudinal fetal neurosonography, including CNS biometry and morphological assessment, together with Doppler evaluation of the umbilical artery and middle cerebral artery to assess placental–fetal hemodynamics and possible fetal redistribution under maternal thermal stress. Validated automated or semi-automated tools may improve the reproducibility of fetal-brain measurements; however, reproducibility may differ substantially among individual advanced fetal imaging parameters, and measurement reliability should therefore be validated on a parameter-by-parameter basis rather than assumed to be uniform across imaging outputs [28,29].

A major strength of this review is the integration of human observational, animal experimental, and organoid evidence, allowing both epidemiological and mechanistic interpretation. Another strength is the specific focus on the potential placenta–brain axis rather than neurodevelopmental outcomes alone. The revised synthesis further strengthens the review by distinguishing direct heat-related evidence from indirect non-heat mechanistic evidence, separating major exposure categories, and analyzing congenital CNS anomalies separately from later neurodevelopmental outcomes.

However, the evidence remains heterogeneous in exposure definition, gestational timing, outcome assessment, and adjustment for confounders. Many human studies relied on ambient-temperature estimates or maternal report rather than individual-level physiological heat measurements [20,21,22,23,24]. Residual confounding by seasonality, air pollution, socioeconomic status, maternal health, infection, fever, and postnatal heat exposure remains possible [3,4,5,6,22,23,24,27].

Ambient-temperature assignment based on residential location may not reflect individual exposure, particularly when information on residential mobility, indoor temperature, cooling access, occupational exposure, hydration, and behavioral adaptation is unavailable. Self-reported fever, sauna, and hot-tub exposure may be affected by recall error and imprecise timing or temperature measurement.

In addition, few human studies included placental biomarkers, inflammatory mediators, or direct biological measures, limiting causal interpretation of the proposed mechanism. Indeed, none of the included human studies formally tested placental mediation. The placenta–brain framework therefore remains dependent on extrapolation from experimental models and indirect mechanistic evidence.

The risk-of-bias profile also varied across studies and should not be summarized uniformly as moderate. Some human studies were affected by serious concerns regarding confounding or exposure misclassification, while animal and organoid studies were frequently limited by incomplete reporting of randomization, allocation concealment, blinding, sample-size justification, biological replication, or litter-level analysis. These limitations reduce the evidential weight of individual findings even when the studies remained eligible for inclusion.

Future studies should use prospective designs with high-resolution exposure assessment, including indoor temperature, air-conditioning access, hydration, occupational heat exposure, personal temperature monitoring, and maternal physiological measures. Gestational-window analyses should be refined, especially for the periconceptional period, early organogenesis, mid-gestation brain development, and late fetal maturation [3,4,5,6,20,21,22,23,24].

Mechanistic studies should further examine placental vascular function, mitochondrial activity, oxidative stress, heat-shock proteins, inflammatory cytokines, glucocorticoid metabolism, and serotonin signaling [7,9,12,13]. Importantly, these pathways should be evaluated within integrated study designs that simultaneously measure maternal thermal exposure, placental molecular responses, fetal brain development, and subsequent child neurodevelopment. Such designs are necessary to distinguish correlation from mediation and to test whether the proposed pathway operates in humans.

Animal studies should improve reporting of randomization, blinding, litter effects, and sample-size justification, while organoid models could be expanded toward placenta–brain or multi-organ systems that better reflect maternal–fetal physiology [9,11,26]. Human studies should distinguish environmental heat from infectious fever and behavioral hyperthermia, and should examine effect modification by maternal comorbidity, socioeconomic vulnerability, occupational exposure, housing quality, and access to cooling.

## 5. Conclusions

This review supports the hypothesis that prenatal thermal exposures may contribute to fetal-brain and offspring neurodevelopmental vulnerability, although the relevant gestational window appears to differ by outcome. The strongest early-pregnancy signal concerns neural tube defects during the periconceptional and neurulation periods, whereas selected studies of later neurodevelopmental outcomes identified susceptibility during early, mid-, or late gestation.

However, the available evidence does not establish a heat-induced placenta–brain axis in humans. Rather, it supports a proposed mechanistic framework derived from partially overlapping epidemiological, animal, and in vitro evidence. Only one included experimental study jointly assessed heat stress, placental alterations, and fetal-brain-related outcomes, while the human studies did not measure placental mediators or conduct mediation analyses.

Ambient heat, infectious fever, behavioral or exogenous heating, experimental hyperthermia, and direct cellular thermal stimulation should also be interpreted as distinct exposures rather than as interchangeable indicators of heat stress. Similarly, neural-tube defects should be distinguished from later fetal-brain and postnatal neurodevelopmental outcomes.

Further prospective, integrated studies are needed to test the proposed mechanistic framework and clarify gestational susceptibility. Until such evidence becomes available, the placenta–brain axis under heat stress should be regarded as a biologically plausible and testable hypothesis rather than an established causal pathway.

## Figures and Tables

**Table 1:** Summary of the characteristics of the included studies.

Study	Country and Design	Exposure Category	Exposure Window	Outcome Domain	Main Finding
Shiota, 1982 [20]	Japan; retrospective human embryo study	Infectious fever/maternal hyperthermia	17–43 days postconception	NTDs	Febrile illness occurred in 14.2% of NTD cases and was more frequent than in control groups; strongest pattern for exencephaly with or without myeloschisis.
Milunsky et al., 1992 [21]	USA; prospective pregnancy cohort	Hot tub, sauna, electric blanket, and fever	First 2–3 months of pregnancy	NTDs	Hot tub, sauna, or fever combined was associated with NTDs (adjusted RR 2.2); hot-tub exposure showed the strongest individual association (adjusted RR 2.8).
Auger et al., 2017 [22]	Canada; retrospective cohort	Ambient outdoor temperature	Weeks 3–4 postconception	NTDs	Maximum weekly temperature was not clearly associated with NTDs; day-specific elevations were observed near the end of week 4 (PR 1.56 and 1.49).
Soim et al., 2017 [23]	USA; population-based case-control study	Weather-related EHEs	Weeks 3–4 postconception	NTDs	No significant overall association or dose-response pattern was observed between EHEs and NTDs.
Guo et al., 2021 [9]	China; experimental mouse study	Gestational heat stress (35 ± 1 °C; 70% RH)	E12.5–E18.5	Placenta and fetal brain	Heat stress disrupted placental glucocorticoid and serotonin-related signaling and increased fetal-brain corticosterone and stress-response gene expression.
Adebiyi et al., 2022 [11]	Nigeria; experimental rat study	Gestational ± postnatal high temperature (40 ± 2 °C)	Gestation to parturition; postnatal extension to P28	Offspring neurodevelopment	Heat exposure altered milestones, behavior, inflammatory and oxidative markers, and MBP expression; effects were more persistent with combined gestational and postnatal exposure.
LaPointe et al., 2024 [24]	USA; matched case-control study	Extreme apparent ambient heat	Eight-week periconception period	NTDs	Increasing consecutive days of extreme heat showed a dose-response association with NTDs; ≥6 days was associated with aOR 1.29, mainly driven by spina bifida.
Lin et al., 2025 [3]	China; birth cohort	Heat waves	Trimester-specific	Neurodevelopmental delay	Heat-wave exposure was associated with increased risk, particularly in trimesters 1 and 3; trimester-1 ORs ranged from 1.186 to 1.383.
DeIngeniis et al., 2025 [25]	USA; longitudinal imaging pilot study	Prenatal extreme heat and Superstorm Sandy co-exposure	Any trimester	Child brain morphology	Extreme heat alone was not associated with basal ganglia volume, but it modified associations between prenatal disaster exposure and selected basal ganglia volumes.
Xu et al., 2025 [26]	China; human brain-organoid study	Periodic heating to 40 °C	Days 10–14 or 20–24 of organoid development	Early neural development	Heating reduced organoid size and neural-tube-like development, reduced progenitor proliferation, increased apoptosis, and altered WNT/BMP/TGF-β signaling.
Barbalat et al., 2025 [4]	France; prospective birth cohort	Ambient temperature, especially night-time heat	Weekly prenatal exposure; key window weeks 14–19	Language development	Severe night-time heat during weeks 14–19 was associated with a 3.2% lower vocabulary production score at age 2 (RR 0.968).
Luglio et al., 2026 [5]	USA; retrospective birth cohort	Extreme night-time and daytime temperature	Weeks 1–37	ASD	High night-time temperature was associated with increased ASD risk during early and late pregnancy; daytime maximum temperature was not significantly associated.
Zhuo et al., 2026 [6]	USA; nested case-control study	High ambient temperature	Gestational weeks 0–31; key window weeks 0–3	CP	Each 5 °C increase during weeks 0–3 was associated with OR 1.08; extreme heat during this window was associated with OR 1.18.
Cai et al., 2026 [27]	China; nationwide preschool cohort	Prenatal and postnatal ambient heat	Entire pregnancy and birth to age 3	Suspected developmental delay	Prenatal moderate and extreme heat were associated with higher odds of SDD (OR 1.28 and 1.35); postnatal associations were stronger.

Abbreviations: aOR, adjusted odds ratio; ASD, autism spectrum disorder; BMP, bone morphogenetic protein; CP, cerebral palsy; E, embryonic day; EHE, extreme heat event; MBP, myelin basic protein; NTD, neural tube defect; OR, odds ratio; P, postnatal day; PR, prevalence ratio; RH, relative humidity; RR, relative risk; SDD, suspected developmental delay; TGF-β, transforming growth factor beta; WNT, Wingless-related integration site signaling pathway.

## Data Availability

No new data were created or analyzed in this study. Data sharing does not apply to this article.

## Supplementary Materials

The following supporting information can be downloaded at: https://www.mdpi.com/article/10.3390/cells15171621/s1, Supplementary Table S1. Deviations from the Registered PROSPERO Protocol; Supplementary Table S2. Complete Reproducible Search Strategies; Supplementary Table S3. Risk-of-Bias Assessment of the Included Studies; Supplementary Table S4. Detailed Characteristics of the Included Studies.
