# Supplementary material for: Placenta–Brain Axis Under Heat Stress: Inflammatory Pathways Linking Prenatal Thermal Exposure to Offspring Neurodevelopment
Source: Cells. 2026 Sep 7;15(17):1621. doi: 10.3390/cells15171621 (PMC13565313; doi:10.3390/cells15171621)
Supplement: Supplementary file 1 [file cells-15-01621-s001.zip › Supplementary_Table_S1 (r).pdf]

Supplementary Table S1. Deviations from the Registered PROSPERO Protocol

Protocol element	Registered protocol	Final manuscript method	Type of deviation	Rationale and likely effect
Bibliographic databases	Google Scholar, MEDLINE, PubMed, and Scopus.	PubMed/MEDLINE, Scopus, Google Scholar, Web of Science, and Embase were searched.	Expansion of information sources	Web of Science and Embase were added to improve coverage and reduce the probability of missing relevant epidemiological, animal, and mechanistic studies. This change increased search sensitivity without narrowing eligibility.
Search logic	The objectives and eligibility criteria allowed studies addressing prenatal heat in relation to placental mechanisms or fetal/offspring neurodevelopment.	Two complementary search streams were used: pregnancy AND heat AND placental/mechanistic terms; and pregnancy AND heat AND fetal-brain/neurodevelopmental terms.	Correction and clarification of search implementation	The original manuscript described all four concept groups as connected by AND, which was more restrictive than the registered eligibility framework. Parallel searches were adopted to align the search with the protocol and reduce the risk of missing studies that evaluated only one of the two outcome domains.
Additional identification methods	Reference-list checking and contacting authors or experts were planned.	Reference lists of eligible articles and relevant reviews were screened; authors or experts were not contacted for additional studies.	Omission of a planned supplementary search method	Author/expert contact was not undertaken. This may have reduced the opportunity to identify additional published or difficult-to-locate studies; the limitation is reported transparently.
Exposure eligibility and categorization	Ambient heat, heat waves, high temperature, maternal hyperthermia, experimental gestational heat stress, thermal stimulation, and other heat-related maternal environmental exposures were eligible.	Exposures were separated into ambient environmental heat/heat waves, infectious fever, behavioral or exogenous heating (e.g., sauna, hot tub, or electric blanket), experimental maternal hyperthermia, and direct cellular or organoid thermal stimulation.	Operational expansion and prespecified subgroup refinement	The protocol included maternal hyperthermia broadly but did not explicitly distinguish infectious fever or behavioral heat sources. These exposures were retained because they were represented in the eligible literature but were analyzed separately, as fever is confounded by infection and inflammation and the exposure mechanisms are not biologically equivalent.

Outcome eligibility and grouping	Main outcomes comprised offspring neurodevelopment, fetal-brain molecular or structural outcomes, and placental inflammatory or stress-response outcomes.	Congenital central nervous system anomalies, particularly neural tube defects, were included and synthesized separately from fetal-brain biometry/morphology and postnatal neurodevelopmental or neurological outcomes.	Outcome expansion and analytical reclassification	Neural tube defects were identified in the eligible literature but were not explicitly listed in the registered outcomes. They were retained as early developmental CNS outcomes and separated from later neurodevelopmental phenotypes to avoid clinically inappropriate aggregation.
Eligible study designs	The record stated that only nonrandomized study types would be included, while also specifying pregnant animal models and experimental placental, fetal-brain, and brain-organoid models as eligible populations.	Human observational studies, animal experiments, and in vitro or organoid mechanistic studies were included and appraised separately.	Clarification of an internally inconsistent protocol field	The final approach followed the protocol's population and mechanistic eligibility statements rather than the generic 'nonrandomized studies only' field. Separate synthesis and appraisal by design preserved methodological distinctions.
Risk-of-bias assessment	The Newcastle-Ottawa Scale was planned for risk-of-bias or quality assessment.	Human exposure studies were assessed with ROBINS-E, animal studies with SYRCLE, and in vitro/organoid studies with a modified OHAT approach supplemented by ToxRTool criteria.	Change of appraisal tools	A single Newcastle-Ottawa assessment was not suitable for the heterogeneous human, animal, and mechanistic evidence base. Design-specific tools were adopted to assess relevant bias domains more appropriately. Eligibility was kept separate from the overall risk-of-bias judgment.
Evidence synthesis structure	Five evidence streams were planned, including human heat-neurodevelopment studies, heat-placenta studies, animal studies, mechanistic placenta-brain studies, and integrated heat-placenta-brain studies.	The synthesis retained separate human and experimental evidence but additionally distinguished direct heat-related evidence from indirect non-heat mechanistic evidence and organized findings by exposure category and outcome domain.	Refinement of the narrative synthesis	The refinement was introduced because only one included study jointly assessed heat stress, placental changes, and fetal-brain outcomes. It prevents indirect mechanistic studies from being interpreted as direct proof of a heat-induced placenta-brain pathway.
Conceptual interpretation	The protocol proposed examination of the placenta as a possible biological mediator and referred to a placenta-brain axis under prenatal heat stress.	The placenta-brain axis was presented as a proposed mechanistic hypothesis rather than an established causal pathway.	Interpretive refinement	Human studies did not measure placental mediators or perform mediation analyses, and direct integrated evidence was limited to one experimental study. The revised wording reduces causal overstatement without changing the review question.

Abbreviations: OHAT, Office of Health Assessment and Translation; PROSPERO, International Prospective Register of Systematic Reviews; ROBINS-E, Risk Of Bias In Non-randomized Studies of Exposures; SYRCLE, Systematic Review Centre for Laboratory Animal Experimentation; ToxRTool, Toxicological Data Reliability Assessment Tool.
