# Supplementary material for: Placenta–Brain Axis Under Heat Stress: Inflammatory Pathways Linking Prenatal Thermal Exposure to Offspring Neurodevelopment
Source: Cells. 2026 Sep 7;15(17):1621. doi: 10.3390/cells15171621 (PMC13565313; doi:10.3390/cells15171621)
Supplement: Supplementary file 1 [file cells-15-01621-s001.zip › Supplementary_Table_S2 (r).pdf]

Supplementary Table S2. Complete Reproducible Search Strategies

Database / platform	Search fields	Exact search strategy	Search date	Records retrieved
PubMed/MEDLINE (PubMed)	MeSH terms and Title/Abstract	Stream 1: ("Pregnancy"[Mesh] OR pregnan*[Title/Abstract] OR prenatal[Title/Abstract] OR antenatal[Title/Abstract] OR gestation*[Title/Abstract] OR maternal exposure[Title/Abstract] OR in utero[Title/Abstract]) AND ("Heat Stress Disorders"[Mesh] OR "Hot Temperature"[Mesh] OR heat stress[Title/Abstract] OR heatwave*[Title/Abstract] OR "heat wave*"[Title/Abstract] OR extreme heat[Title/Abstract] OR ambient temperature[Title/Abstract] OR high temperature[Title/Abstract] OR hyperthermia[Title/Abstract] OR thermal stress[Title/Abstract]) AND ("Placenta"[Mesh] OR placenta*[Title/Abstract] OR placental function[Title/Abstract] OR placental barrier[Title/Abstract] OR maternal-fetal interface[Title/Abstract] OR maternal foetal interface[Title/Abstract] OR inflammation[Title/Abstract] OR cytokine*[Title/Abstract] OR immune activation[Title/Abstract] OR oxidative stress[Title/Abstract] OR heat shock protein*[Title/Abstract] OR stress-response pathway*[Title/Abstract])<br><br>Stream 2: ("Pregnancy"[Mesh] OR pregnan*[Title/Abstract] OR prenatal[Title/Abstract] OR antenatal[Title/Abstract] OR gestation*[Title/Abstract] OR maternal exposure[Title/Abstract] OR in utero[Title/Abstract]) AND ("Heat Stress Disorders"[Mesh] OR "Hot Temperature"[Mesh] OR heat stress[Title/Abstract] OR heatwave*[Title/Abstract] OR "heat wave*"[Title/Abstract] OR extreme heat[Title/Abstract] OR ambient temperature[Title/Abstract] OR high temperature[Title/Abstract] OR hyperthermia[Title/Abstract] OR thermal stress[Title/Abstract]) AND ("Brain"[Mesh] OR "Neurodevelopmental Disorders"[Mesh] OR fetal brain[Title/Abstract] OR foetal brain[Title/Abstract] OR brain development[Title/Abstract] OR brain morphology[Title/Abstract] OR neurodevelopment*[Title/Abstract] OR developmental delay[Title/Abstract] OR child development[Title/Abstract] OR cognition[Title/Abstract] OR language development[Title/Abstract] OR behavio?r[Title/Abstract] OR autism spectrum disorder[Title/Abstract] OR cerebral palsy[Title/Abstract])	30 June 2026	214

Scopus (Elsevier)	TITLE-ABS-KEY	Stream 1: TITLE-ABS-KEY(pregnan* OR prenatal OR antenatal OR gestation* OR "maternal exposure" OR "in utero") AND TITLE-ABS-KEY("heat stress" OR heatwave* OR "heat wave*" OR "extreme heat" OR "ambient temperature" OR "high temperature" OR hyperthermia OR "thermal stress") AND TITLE-ABS-KEY(placenta* OR "placental function" OR "placental barrier" OR "maternal-fetal interface" OR "maternal foetal interface" OR inflammation OR cytokine* OR "immune activation" OR "oxidative stress" OR "heat shock protein*" OR "stress-response pathway*")<br><br>Stream 2: TITLE-ABS-KEY(pregnan* OR prenatal OR antenatal OR gestation* OR "maternal exposure" OR "in utero") AND TITLE-ABS-KEY("heat stress" OR heatwave* OR "heat wave*" OR "extreme heat" OR "ambient temperature" OR "high temperature" OR hyperthermia OR "thermal stress") AND TITLE-ABS-KEY("fetal brain" OR "foetal brain" OR "brain development" OR "brain morphology" OR neurodevelopment* OR "developmental delay" OR "child development" OR cognition OR "language development" OR behavio?r OR "autism spectrum disorder" OR "cerebral palsy")	30 June 2026	386
Web of Science Core Collection (Clarivate)	Topic (TS)	Stream 1: TS=(pregnan* OR prenatal OR antenatal OR gestation* OR "maternal exposure" OR "in utero") AND TS=("heat stress" OR heatwave* OR "heat wave*" OR "extreme heat" OR "ambient temperature" OR "high temperature" OR hyperthermia OR "thermal stress") AND TS=(placenta* OR "placental function" OR "placental barrier" OR "maternal-fetal interface" OR "maternal foetal interface" OR inflammation OR cytokine* OR "immune activation" OR "oxidative stress" OR "heat shock protein*" OR "stress-response pathway*")<br><br>Stream 2: TS=(pregnan* OR prenatal OR antenatal OR gestation* OR "maternal exposure" OR "in utero") AND TS=("heat stress" OR heatwave* OR "heat wave*" OR "extreme heat" OR "ambient temperature" OR "high temperature" OR hyperthermia OR "thermal stress") AND TS=("fetal brain" OR "foetal brain" OR "brain development" OR "brain morphology" OR neurodevelopment* OR "developmental delay" OR "child development" OR cognition OR "language development" OR behavio?r OR "autism spectrum disorder" OR "cerebral palsy")	30 June 2026	222

Embase (Elsevier Embase.com)	Emtree and title/abstract/keyword	Stream 1: ('pregnancy'/exp OR pregnan*:ti,ab,kw OR prenatal:ti,ab,kw OR antenatal:ti,ab,kw OR gestation*:ti,ab,kw OR 'maternal exposure':ti,ab,kw OR 'in utero':ti,ab,kw) AND ('heat stress'/exp OR 'high temperature'/exp OR 'heat stress':ti,ab,kw OR heatwave*:ti,ab,kw OR 'heat wave*':ti,ab,kw OR 'extreme heat':ti,ab,kw OR 'ambient temperature':ti,ab,kw OR 'high temperature':ti,ab,kw OR hyperthermia:ti,ab,kw OR 'thermal stress':ti,ab,kw) AND ('placenta'/exp OR placenta*:ti,ab,kw OR 'placental function':ti,ab,kw OR 'placental barrier':ti,ab,kw OR 'maternal-fetal interface':ti,ab,kw OR inflammation:ti,ab,kw OR cytokine*:ti,ab,kw OR 'immune activation':ti,ab,kw OR 'oxidative stress':ti,ab,kw OR 'heat shock protein*':ti,ab,kw OR 'stress-response pathway*':ti,ab,kw)<br><br>Stream 2: ('pregnancy'/exp OR pregnan*:ti,ab,kw OR prenatal:ti,ab,kw OR antenatal:ti,ab,kw OR gestation*:ti,ab,kw OR 'maternal exposure':ti,ab,kw OR 'in utero':ti,ab,kw) AND ('heat stress'/exp OR 'high temperature'/exp OR 'heat stress':ti,ab,kw OR heatwave*:ti,ab,kw OR 'heat wave*':ti,ab,kw OR 'extreme heat':ti,ab,kw OR 'ambient temperature':ti,ab,kw OR 'high temperature':ti,ab,kw OR hyperthermia:ti,ab,kw OR 'thermal stress':ti,ab,kw) AND ('brain development'/exp OR 'neurodevelopmental disorder'/exp OR 'fetal brain':ti,ab,kw OR 'foetal brain':ti,ab,kw OR 'brain development':ti,ab,kw OR 'brain morphology':ti,ab,kw OR neurodevelopment*:ti,ab,kw OR 'developmental delay':ti,ab,kw OR 'child development':ti,ab,kw OR cognition:ti,ab,kw OR 'language development':ti,ab,kw OR behavio?r:ti,ab,kw OR 'autism spectrum disorder':ti,ab,kw OR 'cerebral palsy':ti,ab,kw)	30 June 2026	248
Google Scholar	Full-text relevance-ranked search	Query 1: ("pregnancy" OR prenatal OR gestational) ("heat stress" OR heatwave OR "extreme heat" OR hyperthermia) (placenta OR placental OR inflammation OR "oxidative stress")<br><br>Query 2: ("pregnancy" OR prenatal OR gestational) ("heat stress" OR heatwave OR "extreme heat" OR hyperthermia) ("fetal brain" OR neurodevelopment OR "developmental delay" OR autism OR "cerebral palsy")<br><br>Results were sorted by relevance. The first 200 results from each query were screened.	30 June 2026	108
