# Supplementary material for: Placenta–Brain Axis Under Heat Stress: Inflammatory Pathways Linking Prenatal Thermal Exposure to Offspring Neurodevelopment
Source: Cells. 2026 Sep 7;15(17):1621. doi: 10.3390/cells15171621 (PMC13565313; doi:10.3390/cells15171621)
Supplement: Supplementary file 1 [file cells-15-01621-s001.zip › Supplementary_Table_S3 (r).pdf]

Supplementary Table S3. Risk-of-Bias Assessment of the Included Studies

Study	Study design/model	Tool	Confounding/ comparability	Selection/ allocation	Exposure assessment	Outcome assessment	Missing data/ reporting	Overall risk of bias	Main justification
Shiota, 1982 [20]	Human retrospective embryo study	ROBINS-E	Serious	Moderate	Serious	Moderate	Moderate	High risk of bias	Self-reported fever/hyperthermia; no precise temperature or duration; no multivariable adjustment, although controls came from the same embryo collection.
Milunsky et al., 1992 [21]	Human prospective follow-up cohort	ROBINS-E	Moderate	Moderate	Moderate	Moderate	Low	Some concerns	Large prospective dataset with good follow-up and some adjustment; exposure relied on maternal report and residual confounding by fever, infection, and behavior remained possible.
Auger et al., 2017 [22]	Human retrospective cohort	ROBINS-E	Moderate	Moderate	Moderate	Moderate	Low	Some concerns	Very large population-based cohort with extensive adjustment; regional meteorological assignment created potential spatial exposure misclassification.
Soim et al., 2017 [23]	Human population-based case-control study	ROBINS-E	Moderate	Low	Moderate	Low	Moderate	Some concerns	Strong surveillance-based case ascertainment and standardized review; ambient proxies may not represent personal heat exposure, and residual confounding remained possible.
LaPointe et al., 2024 [24]	Human matched case-control study	ROBINS-E	Moderate	Moderate	Moderate	Moderate	Low	Some concerns	Matched design and dose-response analysis strengthened the study; vital-record ascertainment and county-level exposure assignment may have introduced misclassification.
Lin et al., 2025 [3]	Human birth cohort	ROBINS-E	Moderate	Low	Moderate	Low	Low	Some concerns	Large cohort, trained outcome assessment, and sensitivity analyses for air pollution and postnatal heat; modeled exposure and residual socioeconomic or environmental confounding remained concerns.
DeIngeniis et al., 2025 [25]	Human longitudinal imaging pilot study	ROBINS-E	Serious	Serious	Serious	Moderate	Moderate	High risk of bias	Very small sample, co-exposure to disaster-related prenatal stress, and limited trimester-specific heat characterization created serious residual-confounding and selection concerns.
Barbalat et al., 2025 [4]	Human prospective birth cohort	ROBINS-E	Moderate	Moderate	Moderate	Moderate	Moderate	Some concerns	High-resolution exposure modeling and extensive adjustment, including air pollutants; attrition, missingness, and parent-reported language assessment may have affected validity.
Luglio et al., 2026 [5]	Human retrospective birth cohort	ROBINS-E	Moderate	Low	Moderate	Moderate	Low	Some concerns	Very large electronic-record cohort with geocoded exposure and multiple sensitivity analyses; diagnostic coding, residual confounding, and personal-exposure misclassification remained possible.
Zhuo et al., 2026 [6]	Human nested case-control study with sibling analysis	ROBINS-E	Moderate	Low	Moderate	Low	Low	Some concerns	Large population-based design and sibling analysis reduced confounding concerns; exposure was based on residence at birth and modeled ambient temperature rather than personal exposure.
Cai et al., 2026 [27]	Human national preschool cohort/cross-sectional analysis	ROBINS-E	Serious	Moderate	Moderate	Moderate	Moderate	High risk of bias	Large sample and broad adjustment; retrospective exposure assignment, cross-sectional developmental assessment, and parent-completed ASQ-3 increased risk of bias.
Guo et al., 2021 [9]	Animal experimental study	SYRCLE	Moderate	Moderate	Low	Moderate	Moderate	Some concerns	Controlled heat protocol and defined gestational window; small groups, pooled RNA-seq samples, and limited reporting of allocation concealment and blinding increased uncertainty.
Adebiyi et al., 2022 [11]	Animal experimental study	SYRCLE	Moderate	Moderate	Low	Moderate	Moderate	Some concerns	Defined heat exposure and multiple neurodevelopmental and biochemical endpoints; possible litter effects, variable sample sizes, and incomplete reporting of randomization and blinding limited confidence.
Xu et al., 2025 [26]	Human brain-organoid mechanistic study	Modified OHAT/ToxRTool	Not applicable	Moderate	Low	Low	Moderate	Some concerns	Two pluripotent stem-cell lines, defined heating windows, and multiple assays supported reliability; organism-level generalizability was limited and blinding/reporting details were incomplete.

Abbreviations: ASQ-3, Ages and Stages Questionnaires, Third Edition; OHAT, Office of Health Assessment and Translation; ROBINS-E, Risk Of Bias In Non-randomized Studies of Exposures; RNA-seq, RNA sequencing; SYRCLE, Systematic Review Centre for Laboratory Animal Experimentation; ToxRTool, Toxicological Data Reliability Assessment Tool.
