# Supplementary material for: Placenta–Brain Axis Under Heat Stress: Inflammatory Pathways Linking Prenatal Thermal Exposure to Offspring Neurodevelopment
Source: Cells. 2026 Sep 7;15(17):1621. doi: 10.3390/cells15171621 (PMC13565313; doi:10.3390/cells15171621)
Supplement: Supplementary file 1 [file cells-15-01621-s001.zip › Supplementary_Table_S4 (r).pdf]

Supplementary Table S4. Detailed Characteristics of the Included Studies

Author, year	Country	Study design	Human / animal / organoid model	Sample size	Exposure definition	Gestational timing of exposure	Outcome type	Outcome measurement tool	Main results	Adjustment / confounders	Authors' conclusion
Shiota, 1982 [20]	Japan	Retrospective epidemiological study using human embryo data	Human embryo population	113 embryos with neural tube defects; controls included 113 gravidity-matched normal embryos, 107 holoprosencephalic embryos, and 121 polydactylous embryos	Maternal hyperthermia defined as reported maternal febrile illness during the index pregnancy. Reported illnesses included cold/flu-like illness, tonsillitis/pharyngitis, rubella, appendicitis, vaginitis, epidemic kerato-conjunctivitis and hyperthyroidism. Precise body temperature and sauna/hot-tub exposure were not available.	Early pregnancy, particularly the critical period of neural tube closure. In 8 of 10 cases with known fever timing, illness occurred during the clinically determined critical period for neural tube closure, 17-43 days after conception.	Neural tube defects in embryos, specifically exencephaly and myeloschisis. Exencephaly was considered the embryologic forerunner of anencephaly, and myeloschisis the forerunner of spina bifida.	Embryological examination of human embryos from the Congenital Anomaly Research Center, Kyoto University; maternal medical/reproductive history collected by obstetricians using a standard interview form.	Maternal febrile illness was reported in 16/113 NTD cases (14.2%). Febrile illness was more frequent in exencephaly +/- myeloschisis (9/50; 18.0%) than in myeloschisis alone (7/63; 11.1%). Control frequencies were lower: 7/113 (6.2%) in gravidity-matched normal controls, 4/107 (3.7%) in holoprosencephaly controls, and 9/121 (7.4%) in polydactyly controls. The difference was significant for all NTD versus gravidity-matched controls (P=0.038) and holoprosencephaly controls (P=0.006). The association was strongest and most consistent for exencephaly +/- myeloschisis.	No multivariable adjustment was reported. The study used comparison groups from the same embryo collection, including gravidity-matched normal controls and embryos with holoprosencephaly or polydactyly. Fisher exact test was used for comparisons. The author notes that previous analysis of the same embryo population did not detect associations between NTD and parental age, consanguinity, occupation, drug use, smoking or drinking habits.	The findings support the hypothesis that maternal hyperthermia in early pregnancy may cause human neural tube defects, especially exencephaly/anencephaly. The author concluded that pregnant women should avoid hyperthermia, particularly in early gestation.

Milunsky et al., 1992 [21]	USA; mainly New England, with participants from Boston, elsewhere in Massachusetts, other New England states and other US states.	Prospective follow-up study of pregnancy outcomes in a cohort of women receiving prenatal care, serum alpha-fetoprotein screening or amniocentesis.	Human pregnancy cohort.	23,491 pregnant women were interviewed. Complete exposure and outcome information was available for 97% of women. After exclusions for missing heat-exposure or outcome data, analyses included 49 pregnancies with neural tube defects.	Maternal heat exposure in early pregnancy: hot tub use, sauna use, electric blanket use and fever. Fever was defined as >=100°F. Analyses also evaluated combined exposure to hot tub, sauna or fever, and number of different heat sources.	Hot tub, sauna and electric blanket exposure were assessed during the first 2 months of pregnancy. Fever was assessed during the first 3 months of pregnancy. The authors emphasized that neural tube closure occurs within the first 6 menstrual weeks of pregnancy.	Central nervous system congenital malformations: neural tube defects.	Pregnancy outcome information was obtained from delivering physicians or mothers. NTDs were defined as spina bifida, anencephaly or encephalocele, alone or in combination with other defects. Relative risks were estimated from crude data and logistic regression.	Any heat exposure was associated with crude RR 1.6 (95% CI 0.9-2.9). Exposure to hot tub, sauna or fever combined was associated with crude RR 2.2 (95% CI 1.2-4.1) and adjusted RR 2.2 (95% CI 1.2-4.2). Individual adjusted RRs were: hot tub 2.8 (95% CI 1.2-6.5), sauna 1.8 (95% CI 0.4-7.9), fever 1.8 (95% CI 0.8-4.1), and electric blanket 1.2 (95% CI 0.5-2.6). Risk increased with exposure to two heat sources: RR 6.2 (95% CI 2.2-17.2).	Adjusted for maternal age, folic acid supplementation, family history of neural tube defects and exposure to other heat sources.	Exposure to heat in the form of hot tub, sauna or fever during the first trimester of pregnancy was associated with an increased risk of neural tube defects. Hot tub exposure appeared to have the strongest effect, whereas electric blanket exposure was not materially associated with increased NTD risk.
Auger et al., 2017 [22]	Canada; Quebec	Retrospective cohort study	Human fetuses/live births	887,710 fetuses in utero during the critical exposure window; 297 neural tube defects identified	Elevated ambient outdoor temperature, assessed mainly as maximum daily temperature and maximum weekly temperature. Weather data were obtained from 18 meteorological stations representative of Quebec health regions. Temperature was analysed continuously relative to 20°C and categorically as <20°C, 20–24.9°C, 25–29.9°C and ≥30°C.	Third and fourth weeks postconception, corresponding to early central nervous system formation and neural tube closure. The main critical window was the end of the fourth week postconception, when neural tube closure occurs. Study months were April to September, 1988–2012.	Neural tube defects, including spina bifida and anencephalus/encephalocoele. Other central nervous system defects, such as microcephalus and congenital hydrocephalus, were analysed as a comparison group.	Hospital discharge abstracts for mothers and live-born infants in Quebec; NTD diagnoses documented on neonatal discharge charts.	Overall NTD prevalence was 33.5 per 100,000 live births. Maximum weekly temperature during weeks 3–4 postconception was not clearly associated with NTDs. In day-specific analyses, exposure to 30°C versus 20°C was associated with increased NTD risk on day 5 of week 4 postconception, PR 1.56, 95% CI 1.04–2.35, and day 6 of week 4, PR 1.49, 95% CI 1.00–2.21. The trend was apparent for spina bifida and anencephalus/encephalocoele. No association was observed during week 3 postconception or with other CNS defects.	Adjusted for humidity, maternal age, multiple birth, maternal comorbidity, material deprivation, parity, month of conception and year of conception. Comorbidities included pre-existing diabetes, hypertensive heart disease or obesity, blood, thyroid, epilepsy or mood disorders, connective tissue diseases, tobacco or substance abuse.	The authors concluded that elevated ambient temperatures may be weakly associated with increased risk of neural tube defects during the period of neural tube closure, particularly toward the end of the fourth week postconception. They emphasized that the findings require follow-up studies with more precise exposure timing and temperature measurement.

Soim et al., 2017 [23]	USA; National Birth Defects Prevention Study sites in Arkansas, California, Georgia, Iowa, New York, North Carolina, Texas and Utah	Population-based case-control study	Human pregnancies; infants/fetuses with and without major birth defects	Final analytic sample: 326 neural tube defect cases and 1,781 controls. Cases included 210 spina bifida, 81 anencephaly/craniorachischisis and 35 encephalocele cases.	Weather-related extreme heat events (EHEs), based on daily universal apparent maximum temperature (UATmax), which combines temperature, humidity and wind speed. EHE95 was defined as at least 2 consecutive days with daily UATmax above the 95th percentile for season, year and weather station. EHE90 was defined as at least 3 consecutive days with daily UATmax above the 90th percentile.	Critical neural-tube-closure window: third and fourth weeks post-conception. Only pregnancies with at least one day of this window during summer months, June-August, were included.	Neural tube defects, including spina bifida, anencephaly/craniorachischisis and encephalocele.	NTD cases were identified through birth-defect surveillance systems in the National Birth Defects Prevention Study. Medical records were reviewed by clinical geneticists using standardized diagnostic criteria. Controls were singleton live-born infants without major birth defects.	Overall, no statistically significant association was observed between EHEs and NTDs. For the full NBDPS sample, EHE95 was associated with aOR 0.93, 95% CI 0.71-1.21, and EHE90 with aOR 0.82, 95% CI 0.62-1.07. At climate-region level, some estimates were elevated but not statistically significant, particularly EHE95 in New York/Northeast, North Carolina-Georgia/Southeast and Iowa/Upper Midwest. No dose-response relationship was observed for EHE frequency or duration. No effect modification by maternal BMI was found.	Final models adjusted for maternal age, maternal race/ethnicity and pre-gestational BMI. Potential confounders evaluated included maternal education, parity, prenatal care, folic acid use, fever, family history of NTDs, diuretic/laxative use, caffeine, alcohol, smoking, secondhand smoke, occupation with potential heat exposure and residential history. Sub-analyses considered no fever, no diuretic use, complete residence history, live births only, distance from weather station and NTD subtype.	The authors concluded that weather-related extreme heat events during the relevant embryologic window did not appear to appreciably affect the risk of NTDs. They recommended future studies with improved exposure assessment, including personal temperature monitoring and better accounting for maternal behaviours that may modify heat exposure.

Guo et al., 2021 [9]	China	Experimental animal study	Pregnant ICR mice and fetuses	Pregnant mice: thermal-neutral group n=11; heat-stress group n=8. At E18.5, placenta, fetal brain, fetal duodenum and fetal jejunum were collected. RNA-seq used pooled biological replicates: placenta n=2/group, fetal brain n=2/group, fetal duodenum n=3/group and fetal jejunum n=3/group; biochemical and protein assays used variable sample sizes depending on tissue/marker.	Maternal heat stress during late pregnancy. Thermal-neutral group: 24 +/- 1 deg C and 50% relative humidity. Heat-stress group: 35 +/- 1 deg C and 70% relative humidity.	Late gestation, from embryonic day E12.5 to E18.5. Pregnant mice were sacrificed at E18.5, and fetal tissues were collected immediately.	Placental barrier and serotonin-system outcomes; fetal brain stress-response outcomes; fetal intestinal nutrient transport/metabolism outcomes; fetal survival.	Placental and fetal tissue analyses including RNA sequencing, GO/KEGG enrichment, PPI/hub-gene analysis, qRT-PCR, Western blotting, biochemical assays for triglycerides and total cholesterol, and fetal brain ACTH/corticosterone ELISA.	Maternal heat stress increased the number/rate of dead fetuses, 9.48% in the heat-stress group vs 0.70% in thermal-neutral controls. Transcriptome analysis showed nutrient transport/metabolism genes were up-regulated in placenta but down-regulated in fetal duodenum and jejunum. Placental triglyceride concentration increased, whereas triglycerides decreased in fetal duodenum and jejunum, and total cholesterol decreased in fetal duodenum. Maternal heat stress reduced placental Hsd11b2 and Htr1d mRNA/protein levels, indicating disruption of the glucocorticoid barrier and serotonin system. In fetal brain, corticosterone increased, and hsp90b1, hyou1 and crhr1 expression, enriched in glucocorticoid/hypoxia responses, was increased.	No observational confounder adjustment. Experimental comparison was between thermal-neutral and heat-stress groups. Pregnant mice were randomly divided into groups. Analyses used independent-sample t-tests for group comparisons. Important design features include controlled temperature/humidity exposure, same gestational window and matched tissue collection at E18.5.	The authors concluded that maternal heat stress disrupted the placental glucocorticoid barrier and serotonin system, associated with increased fetal-brain corticosterone and altered glucocorticoid-response gene expression. They suggested that these alterations may decrease nutrient transport and metabolism in the fetal intestine, while the placenta may adapt nutrient transport/metabolism to meet fetal-growth demands. They further suggested that heat stress during late pregnancy may increase the risk of offspring neurodevelopmental and gastrointestinal disorders.

Adebiyi et al., 2022 [11]	Nigeria	Experimental animal study	Pregnant Wistar rats and offspring pups	40 adult rats initially: 30 female and 10 male Wistar rats. Pregnant dams: control n=10; high-temperature gestational exposure n=20, later divided after parturition into GE and GE+PE groups. Pup numbers reported for birth weight: control n=73 pups, GE n=76 pups, GE+PE n=71 pups. Behavioural testing included approximately n=44 pups/group; brain assays were performed at P7, P14, P21 and P28, with n=5/group/time point for some analyses.	High environmental temperature / high housing temperature. Control dams were kept at standard temperature, approximately 25–27°C or 26 ± 2°C. Heat-exposed pregnant rats were housed at 40 ± 2°C for 5 hours daily to simulate heat-wave exposure.	Pregnancy was detected by palpation around gestational days 5–8; dams were then exposed to 40 ± 2°C for 5 h/day until parturition. After birth, one exposed group returned to standard temperature and another remained heat-exposed until P28	Neurodevelopmental milestones, neonatal reflexes, behaviour, brain inflammatory/oxidative markers, myelination-related markers, neuroarchitecture and gut microbiome changes.	Developmental milestone/reflex tests: eye opening, ear pinnae opening, fur development, surface righting reflex, forelimb suspension, cliff aversion, hindlimb suspension, grasping reflex, negative geotaxis, ambulation and hindfoot angle. Brain analyses: ELISA for IL-4, TNF-alpha, IL-10, SOD and MDA; Western blotting and immunofluorescence for MBP, NeuN, GFAP and IBA1; gut bacterial identification using culture and 16S rRNA PCR/sequencing.	High temperature exposure did not significantly alter pregnancy length, litter size or birth weight. Heat-exposed pups showed neurodevelopmental and behavioural alterations, including increased hindfoot angle, reduced forelimb suspension latency, delayed ear pinnae opening, reduced grasping reflex especially in GE+PE pups, reduced body weight gain and lower survival in GE+PE pups. TNF-α, IL-4, IL-10 and MDA levels were increased in gestational-only and gestational-plus-postnatal heat-exposed pups, while MBP expression was reduced.pups, especially GE+PE. The study also reported altered gut microbiome patterns, including Firmicutes and Proteobacteria differences.	No confounder adjustment as in observational studies. Experimental comparisons were performed between control, GE and GE+PE groups. Statistical analyses used one-way ANOVA with Tukey post hoc tests and two-way ANOVA for cytokines and oxidative-stress markers. Reported controls included standardized animal housing, controlled light/dark cycle, defined heat-exposure protocol and age-matched postnatal assessments.	The authors concluded that exposure to high environmental temperature during gestation and postnatal life delayed neurodevelopmental milestones and altered behaviour, inflammatory cytokines, oxidative-stress biomarkers, MBP expression and gut microbiome composition in rat pups. Gestational-only exposed pups showed some developmental setback but appeared to improve over the course of testing, whereas combined gestational and postnatal exposure produced more persistent adverse effects.

LaPointe et al., 2024 [24]	USA; Georgia	Population-based matched case-control study	Human pregnancies; live births and fetal deaths	825 isolated NTD cases and 3,300 controls matched 1:4. Cases included 473 anencephaly and 352 spina bifida cases.	Extreme ambient heat exposure defined as consecutive days on which daily apparent temperature exceeded the county-specific 95th percentile, based on the 1981–2010 30-year normal. Daily maximum, minimum and apparent temperature metrics were considered; main analysis used apparent temperature. County-level temperature was derived from Daymet 1 km² gridded meteorological data and linked to maternal county of residence.	Eight-week periconception period: four preconception weeks and four postconception weeks. Weekly analyses covered weeks -2 through 6 relative to last menstrual period; weeks 3–6 represented postconception / early pregnancy. This window captures neurulation, approximately 17–28 days post-fertilization.	Isolated neural tube defects, including anencephaly and spina bifida / meningomyelocele.	Georgia birth and fetal death records, 1994–2017. NTD cases were identified from vital records as live births or fetal deaths with diagnosis of anencephaly or spina bifida. Controls were live or stillborn infants without birth defects reported in vital records.	A dose-response association was observed between increasing consecutive days of extreme apparent heat during periconception and odds of NTDs. Compared with no extreme heat exposure, aORs for any NTD were 1.09, 95% CI 1.01–1.17, for 1–2 consecutive days; 1.18, 95% CI 1.03–1.36, for 3–5 days; and 1.29, 95% CI 1.04–1.58, for >=6 days. Exposure to >=3 consecutive days was associated with higher odds of any NTD, aOR 1.24, 95% CI 1.03–1.49. Associations were largely driven by spina bifida; for >=6 days, spina bifida aOR was 1.41, 95% CI 1.03–1.93. Weekly analyses showed consistently elevated odds during periconception, with stronger associations in the postconception period, especially gestational week 5 for spina bifida, aOR 1.84, 95% CI 1.29–2.63.	Conditional logistic regression models adjusted for maternal age, education, ethnicity, and month and year of last menstrual period. Cases and controls were matched on county of residence and time period of delivery, pre- vs post-folic acid fortification. Effect modification was evaluated by race, ethnicity, season of LMP, delivery period and county-level poverty.	The authors concluded that maternal exposure to extreme ambient heat during the periconception period was positively associated with NTDs in offspring, with evidence of a dose-response pattern. They emphasized that increasing global temperatures may pose health threats for pregnant people and may have implications for increased risk of neural tube defects in offspring.

Lin et al., 2025 [3]	China; Foshan, Guangdong Province	Birth cohort study	Human mother-child pairs	67,453 child-mother pairs included in the analytical cohort; 3,805 children (5.64%) were identified as having neurodevelopmental delay	Maternal exposure to heat waves during pregnancy. Heat waves were defined using daily 24-hour mean temperature from CLDAS v2.0, spatial resolution 0.0625° x 0.0625°. Definitions combined intensity thresholds (>=90th, >=92.5th or >=95th percentile of daily mean temperature) with duration (at least 2, 3 or 4 consecutive days), resulting in 9 heat-wave definitions	Trimester-specific exposure: trimester 1, weeks 1-13; trimester 2, weeks 14-28; trimester 3, week 29 to delivery. Exposure was assessed as yes/no heat-wave exposure during each trimester and as number of heat-wave days	Neurodevelopmental delay in young children; individual domains included gross motor, fine motor, language, adaptability and social response	Neuropsychological Examination Scale for Chinese Children (NESCC), administered by trained medical workers. Developmental quotient (DQ) was calculated as full-scale score / child's exact age x 100. Neurodevelopmental delay was defined as DQ <75	Heat-wave exposure during pregnancy was significantly associated with increased risk of neurodevelopmental delay. Associations were most evident for trimester 1 and trimester 3 exposure. For trimester 1, most heat-wave definitions showed increased risk, with ORs ranging from 1.186 to 1.383, except P92.5_D4 and P95_D4. Trimester 3 associations were similar but slightly smaller. Trimester 2 results were mixed. No clear dose-response pattern was observed for number of heat-wave days. Results remained generally stable after sensitivity analyses including additional adjustment for PM2.5 or NO2, exclusion of preterm/low-birth-weight children, and adjustment for postnatal heat-wave exposure	Adjusted for maternal age at delivery, maternal employment status, maternal education, folic acid supplementation during pregnancy, child sex, child age at neurodevelopment assessment, season of conception, delivery mode, preterm birth, low birth weight, feeding pattern during the first 6 months and relative humidity. Sensitivity analyses additionally adjusted for PM2.5 or NO2 and postnatal heat-wave exposure	The authors concluded that prenatal exposure to heat waves, especially during early and late pregnancy, was associated with increased risks of neurodevelopmental delay in young children. They stated that the study provides suggestive evidence that prenatal heat-wave exposure may have detrimental effects on children's neurodevelopment and that further studies are needed to verify the findings

DeIngeniis et al., 2025 [25]	USA; New York City	Longitudinal human cohort / imaging pilot study from the Stress in Pregnancy, SIP, cohort	Human children with prenatal exposure assessment and MRI at school age	34 children, mean age 8.70 years; 11 exposed to Superstorm Sandy in utero and 23 non-exposed. Extreme heat exposure was present in 7/11 Superstorm Sandy-exposed children and 17/23 non-exposed children	Prenatal extreme ambient heat exposure, defined as exposure to at least one day with high temperature >95°F, equivalent to >35°C, during any trimester of pregnancy. Temperature estimates were extracted from three New York City weather stations, Central Park, LaGuardia Airport and John F. Kennedy Airport, and assigned using the station closest to the maternal residential coordinates	Any trimester of pregnancy. The study did not provide trimester-specific heat results; exposure was classified as yes/no exposure to at least one day >95°F during pregnancy	Child brain morphology, specifically basal ganglia gray matter volumes: bilateral caudate, putamen, pallidum and nucleus accumbens. The study also examined interaction between prenatal extreme heat and prenatal Superstorm Sandy exposure	Structural brain MRI at approximately 8 years of age using a Siemens 3 Tesla MRI scanner. T1-weighted MPRAGE images were processed with FreeSurfer version 7.4.1. Regional basal ganglia volumes were normalized by total intracranial volume	Extreme heat alone was not significantly associated with basal ganglia volume. Superstorm Sandy exposure was associated with larger left and right putamen, larger left and right pallidum, and larger right caudate. Interaction models showed that extreme heat moderated the association between Superstorm Sandy exposure and basal ganglia volume: co-exposure was associated with reduced left nucleus accumbens volume and increased left pallidum volume. Specifically, the interaction was significant for left nucleus accumbens, adjusted p=0.05, and left pallidum, adjusted p=0.14 using a 15% false discovery rate threshold	Models adjusted for child age at MRI, child biological sex, maternal age at childbirth, maternal race, prenatal stress class, objective Superstorm Sandy challenge, family socioeconomic status, number of days exposed to >95°F, season of conception and New York City Heat Vulnerability Index. Generalized estimating equations were used to account for intrafamilial correlations among siblings	The authors concluded that prenatal exposure to Superstorm Sandy was associated with altered basal ganglia gray matter volume in school-aged children, and that prenatal extreme heat may amplify the adverse impact of disaster-related prenatal stress on basal ganglia development. They emphasized the need to consider co-occurring climate-related risks during pregnancy when studying long-term child brain development

Xu et al., 2025 [26]	China	Experimental in vitro mechanistic study	Human brain organoids derived from human pluripotent stem cells; ESC line H9 and iPSC line IMR90-4	Multiple organoid batches from H9 and IMR90-4 cell lines. For size analyses: H9 control n=37, D10–14 heating n=37, D20–24 heating n=25; IMR90-4 control n=49, D10–14 heating n=48, D20–24 heating n=55. For neural-tube structure analyses: n=60 organoids/group/cell line. For 3D reconstruction in H9 organoids: control n=16, D10–14 heating n=31, D20–24 heating n=21. RNA-seq used three groups of H9 organoids with at least 15 organoids per group.	Periodic thermal stimulation / periodic heat exposure designed to model maternal fever. Control organoids were maintained at 37°C. Heated organoids were transferred to 40°C twice daily, from 09:30–10:30 and 15:30–16:30.	In vitro developmental timing corresponding to early prenatal neural development. Periodic heating was applied either during day 10–14, representing neural tube formation, or day 20–24, representing a later critical stage of neural tube development. Organoids were harvested and assessed at day 30.	Abnormal prenatal neural development; brain organoid size; neural tube-like structure; neural progenitor proliferation; apoptosis; neural differentiation; transcriptomic changes related to neurodevelopmental disorders.	Bright-field organoid size measurements; immunofluorescence for PKC-λ, SOX2, KI67, PAX6, EdU, TBR1, TBR2 and other markers; tissue clearing and 3D reconstruction; bulk RNA sequencing; GO/KEGG/GSEA and disease-gene analyses; qPCR; TUNEL assay for apoptosis.	Periodic heat exposure reduced brain organoid size and impaired neural tube-like development. D10–14 heating decreased ventricle area, while both D10–14 and D20–24 heating reduced total loop area and lumen volume. RNA-seq showed transcriptomic changes after heating, including activation of WNT, BMP, TGF-β and neural differentiation pathways, up-regulation of apoptosis and reactive oxygen species pathways, and down-regulation of G2/M checkpoint-related signatures. Heat exposure reduced KI67, PAX6, SOX2 and EdU-positive proliferative cells, increased apoptosis, promoted cell-cycle exit, increased differentiative divisions and increased TBR1/TBR2-related neural differentiation markers. Heated organoids also showed transcriptomic similarity to neurodevelopmental-disorder-related organoid models, particularly ASD-related models.	No observational confounder adjustment. Experimental comparison was between control, D10–14 heating and D20–24 heating organoid groups. The authors reported at least three randomized independent experiments. Statistical analyses included one-way ANOVA and chi-square tests. Relevant methodological controls included use of two human pluripotent stem-cell lines, defined heating windows, control temperature exposure and multiple molecular/imaging assays.	The authors concluded that periodic heat exposure can adversely affect early human fetal brain development by reducing organoid size, impairing neural tube development and altering key developmental pathways, especially WNT signalling. They suggested that maternal hyperthermia may contribute to neurodevelopmental disorders through altered proliferation, apoptosis and neural differentiation, and that periodically heated human brain organoids provide a useful model for studying environmental thermal effects on fetal brain development.

Barbalat et al., 2025 [4]	France; national ELFE birth cohort	Prospective national birth cohort study	Human children with prenatal and postnatal temperature exposure assessment	Final analytical sample: 12,163 children from the ELFE cohort. Original cohort included 18,329 children; after excluding multiple pregnancies, loss to follow-up, missing MB-CDI score, incomplete exposure data and high covariate missingness, 12,163 remained.	Ambient temperature exposure estimated at the maternal/child home address using high-resolution spatiotemporal models. Three temperature indicators were used: mean temperature (Tmean) as overall exposure; maximum temperature (Tmax) as daytime exposure; and minimum temperature (Tmin) as night-time exposure. Heat and cold were examined using moderate, severe and extreme percentiles compared with median temperature.	Prenatal exposure was assessed weekly during the first 30 weeks of gestation. The most relevant prenatal heat finding was severe night-time heat, Tmin 15.6°C, 95th percentile, versus 8.3°C median, during gestational weeks 14–19. Postnatal exposure was also assessed during the first 91 weeks after birth, but for the strict prenatal review the prenatal window is the key inclusion-relevant component.	Linguistic development / early neurodevelopmental language outcome at 2 years of age.	Vocabulary production score from the MacArthur-Bates Communicative Development Inventories (MB-CDI). Parents reported whether the child could spontaneously pronounce each word from a list of 100 French words; higher scores indicated better linguistic development.	Prenatal severe night-time heat during gestational weeks 14–19 was associated with lower MB-CDI vocabulary production scores at age 2: RR 0.968, 95% CI 0.939–0.998, corresponding to a 3.2% decrease. Similar negative associations were observed for moderate and extreme night-time heat. Overall and daytime prenatal heat were not clearly associated with lower scores. Postnatal heat showed stronger negative associations, including severe overall heat during weeks 1–28 after birth, RR 0.852, 95% CI 0.756–0.960. Results were broadly consistent after adjustment for air pollutants, PM2.5, PM10 and NO2. No significant sex differences were detected.	Models adjusted for neighborhood socioeconomic context, urbanization, vegetation, parental socioeconomic and demographic factors, number of languages spoken at home, pre-pregnancy medical history including parity, neurodevelopmental disorders and maternal obesity, child age and sex, and parental behaviors related to food/drug exposure, breastfeeding, alcohol and tobacco. Secondary models additionally adjusted for PM2.5, PM10 and NO2. Distributed lag non-linear models were used to identify critical exposure windows.	The authors concluded that hot temperatures during pregnancy and after birth were associated with poorer language acquisition at age 2. They identified the second trimester, especially night-time heat, and the early postnatal months as potential sensitive windows. They suggested that policymakers should consider neurodevelopmental impairment as a possible adverse effect of climate change.

Luglio et al., 2026 [5]	USA; Southern California	Population-based retrospective birth cohort study using Kaiser Permanente Southern California electronic medical records	Human mother-child pairs	294,937 term singleton mother–child pairs; 4,076 autism diagnoses by age 5	Prenatal exposure to extreme heat, assessed as weekly average daily minimum temperature, Tmin, representing night-time temperature, and weekly average daily maximum temperature, Tmax, representing daytime temperature. Temperature estimates were assigned to geocoded maternal residential addresses using the gridMET dataset. Extreme heat was compared at the 90th and 99th percentiles versus the 50th percentile	Gestational weeks 1-37. Sensitive windows were identified for high Tmin during early pregnancy, weeks 1-7 for the 90th percentile and weeks 1-10 for the 99th percentile, and late pregnancy, weeks 32-37 for the 90th percentile and weeks 30-37 for the 99th percentile. Tmax showed no significant sensitive window	Autism spectrum disorder diagnosis by age 5	Autism diagnosis identified from electronic medical records using ICD-9 codes 299.0, 299.1, 299.8 and 299.9 and ICD-10 codes F84.0, F84.3, F84.5, F84.8 and F84.9, requiring diagnosis codes in at least two separate visits. Children were followed until autism diagnosis, death, loss to follow-up, December 31, 2019, or age 5	High night-time temperature, Tmin, was associated with increased autism risk. For the 90th percentile versus median Tmin, sensitive windows were weeks 1-7, cumulative HR 1.085, 95% CI 1.011-1.165, and weeks 32-37, HR 1.074, 95% CI 1.011-1.142. For the 99th percentile versus median Tmin, sensitive windows were weeks 1-10, HR 1.154, 95% CI 1.040-1.288, and weeks 30-37, HR 1.132, 95% CI 1.030-1.246. Across all 37 weeks, cumulative HRs for Tmin were 1.243, 95% CI 1.002-1.542, at the 90th percentile and 1.382, 95% CI 1.062-1.797, at the 99th percentile. No significant association was observed for Tmax. Sex-stratified analyses suggested associations mainly among male children, but sex interaction was not statistically significant	Main Cox proportional hazards distributed lag non-linear models adjusted for birth year, season at last menstrual period, maternal age at delivery, parity, maternal race/ethnicity, maternal education, maternal comorbidities, pre-pregnancy obesity, pre-pregnancy diabetes, census-tract income, child sex and pregnancy-average minimum relative humidity. Sensitivity analyses additionally considered PM2.5, ozone, NDVI, neighborhood disadvantage and inclusion of preterm births in expanded cohorts	The authors concluded that prenatal exposure to high night-time temperatures during early and late pregnancy was associated with increased risk of autism diagnosis in children. They suggested that reducing exposure to extreme night-time heat during pregnancy may help reduce autism risk and emphasized the need for further research into mechanisms and gestational windows of susceptibility

Zhuo et al., 2026 [6]	USA; California	Population-based nested case-control study with sibling comparison analysis.	Human pregnancies / offspring	5,938 CP cases and 1,092,313 controls, selected as a 20% random sample of singleton births without CP.	High ambient temperature during pregnancy, assessed as weekly average daily mean temperature at the maternal residential address at birth. Temperature data came from Daymet v4 with 1 km2 spatial resolution. Main exposure was per 5 deg C increase in weekly ambient temperature. Extreme heat was defined as weekly mean temperature above the location-specific 90th percentile, approximately 24.7 deg C.	Gestational week 0-31 and the final seven weeks before birth. The main susceptible window was early pregnancy, especially gestational weeks 0-3. Cumulative effects were also estimated for trimester 1, weeks 0-13; trimester 2, weeks 14-27; trimester 3, weeks 28-31; and final seven weeks before birth.	Childhood cerebral palsy, a neuromotor neurodevelopmental disorder.	CP cases were identified by probabilistic linkage between California Department of Developmental Services diagnostic records from 2005-2021 and California birth records from 2005-2015. Controls were a 20% random sample of singleton births without CP in California during 2005-2015.	Each 5 deg C increase in weekly ambient temperature during the first four gestational weeks was associated with increased CP risk. Cumulative exposure during weeks 0-3 was associated with OR 1.08, 95% credible interval 1.02-1.15. Extreme heat during weeks 0-3 was associated with OR 1.18, 95% credible interval 1.00-1.43. Cumulative trimester associations were also observed: trimester 1 ambient temperature OR 1.10, 95% CI 1.03-1.17; trimester 1 extreme heat OR 1.43, 95% CI 1.00-2.05; trimester 2 ambient temperature OR 1.08, 95% CI 1.01-1.16. No increased association was seen in trimester 3. Sibling analyses showed a similar early-pregnancy pattern.	Main models adjusted for year of birth, season of conception, birth county, maternal age at delivery, maternal race/ethnicity, maternal education, primary payment source for prenatal care, parity, and census-tract social vulnerability domains including socioeconomic status, household composition/disability, minority status/language, and housing/transportation. Sibling analyses adjusted for year of birth, maternal primary insurance type for prenatal care, maternal age at delivery and parity.	The authors concluded that exposure to high ambient temperature in early pregnancy may contribute to the etiology of childhood cerebral palsy. They emphasized that these findings have implications for monitoring childhood neurodevelopmental disorders in the context of climate change.

Cai et al., 2026 [27]	China; Chinese National Cohort of Motor Development, CNCMD, covering 551 county-level cities	Large nationwide observational study / cross-sectional analysis of a national preschool-child cohort with retrospective early-life exposure assignment	Human preschool children with prenatal and postnatal temperature exposure assessment	101,228 children aged 3-5.5 years; 17,712 had suspected developmental delay, SDD, and 83,516 did not	Early-life ambient heat exposure assessed using daily mean temperature and heatwave indicators. Daily mean temperature, daily maximum temperature and relative humidity were obtained from 699 weather stations across China from 2012-2019 and matched to participants' residential longitude/latitude. Moderate heat was defined as the 85th percentile and extreme heat as the 95th percentile of temperature within each exposure window. Heatwaves were defined using daily maximum temperature above station-specific 90th, 92.5th, 95th or 97.5th percentiles for at least 2, 3 or 4 consecutive days, generating 12 heatwave definitions	Two early-life exposure windows: entire pregnancy, from last menstrual period to birth, and postnatal exposure from birth to 3 years. For the strict prenatal review, the relevant exposure window is the entire pregnancy. Mean prenatal temperature was 15.1 +/- 4.5°C; prenatal moderate heat, 85th percentile, was 19.5°C; prenatal extreme heat, 95th percentile, was 20.7°C	Suspected neurodevelopmental delay overall and in five domains: communication, gross motor, fine motor, problem-solving and personal-social development	Ages and Stages Questionnaires, Third Edition, ASQ-3, Chinese version, completed online by parents. The ASQ-3 assessed five domains with six items each. Domain-specific SDD was defined as a score below mean - 2 SD, and overall SDD was defined as SDD in at least one domain	Prenatal and postnatal heat were associated with higher odds of overall SDD. For entire pregnancy, moderate heat was associated with OR 1.28, 95% CI 1.17-1.39, and extreme heat with OR 1.35, 95% CI 1.22-1.49, compared with the minimum-risk temperature. Postnatal heat showed stronger associations: moderate heat OR 1.49, 95% CI 1.30-1.72, and extreme heat OR 1.53, 95% CI 1.32-1.79. Heat exposure was also associated with SDD across all five developmental domains. For prenatal heatwaves, HW-P95-d2 and HW-P97.5-d2 were significantly associated with increased SDD risk. For postnatal heatwaves, all 12 definitions were associated with increased SDD risk. Associations were robust in sensitivity analyses including adjustment for region, air-conditioner ownership and alternative air-pollution models	Main models adjusted for child sex, age, BMI-for-age z-score, birth weight, gestational weeks, pregnancy complications, delivery mode, breastfeeding, NICU admission, psychiatric medication use, maternal age at conception, maternal education, paternal education, maternal employment, disposable income, family structure, marital status, urban/rural residence, birth season, relative humidity, PM2.5 concentration and random contribution of kindergarten. Sensitivity analyses considered geographical regions, provincial air-conditioner ownership, mutual adjustment for prenatal/postnatal temperature and substitution of PM2.5 with NO2 or O3	The authors concluded that early-life exposure to high ambient temperatures, including both prenatal and postnatal exposure, was associated with increased risk of overall and domain-specific suspected neurodevelopmental delay in Chinese preschool children. They emphasized that the developing brain is vulnerable to heat exposure and that climate change may increase temperature-related neurodevelopmental risks

Abbreviations: ACTH,adrenocorticotropic hormone; ANOVA, analysis of variance; aOR, adjusted odds ratio; ASQ-3, Ages and Stages Questionnaires, Third Edition; ASD, autism spectrum disorder; BMI, body mass index; CI, confidence interval; CLDAS, China Meteorological Administration Land Data Assimilation System; CNCMD, Chinese National Cohort of Motor Development; CNS, central nervous system; CP, cerebral palsy; DLNM, distributed lag non-linear model; DQ, developmental quotient; EHE, extreme heat event; ELFE, Étude Longitudinale Française depuis l'Enfance; ELISA, enzyme-linked immunosorbent assay; ESC, embryonic stem cell; FDR, false discovery rate; GE, gestational exposure; GE+PE, gestational exposure plus postnatal exposure; GFAP, glial fibrillary acidic protein; GO, Gene Ontology; GSEA, gene set enrichment analysis; HR, hazard ratio; HW, heatwave; ICD-9, International Classification of Diseases, Ninth Revision; ICD-10, International Classification of Diseases, Tenth Revision; ICR, Institute of Cancer Research mouse strain; IBA1, ionized calcium-binding adaptor molecule 1; IL, interleukin; iPSC, induced pluripotent stem cell; KEGG, Kyoto Encyclopedia of Genes and Genomes; LMP, last menstrual period; MB-CDI, MacArthur-Bates Communicative Development Inventories; MBP, myelin basic protein; MDA, malondialdehyde; MRI, magnetic resonance imaging; NBDPS, National Birth Defects Prevention Study; NDVI, normalized difference vegetation index; NESCC, Neuropsychological Examination Scale for Chinese Children; NeuN, neuronal nuclear antigen; NICU, neonatal intensive care unit; NO_2, nitrogen dioxide; NTD, neural tube defect; OR, odds ratio; O_3, ozone; $PM_{2.5}$, particulate matter with aerodynamic diameter ≤2.5 μm; PM_{10}, particulate matter with aerodynamic diameter ≤10 μm; PPI, protein–protein interaction; PR, prevalence ratio; qPCR, quantitative polymerase chain reaction; qRT-PCR, quantitative reverse-transcription polymerase chain reaction; RNA-seq, RNA sequencing; RR, relative risk/risk ratio; SDD, suspected developmental delay; SIP, Stress in Pregnancy cohort; SOD, superoxide dismutase; TGF-β, transforming growth factor beta; Tmax, maximum temperature; Tmean, mean temperature; Tmin, minimum temperature; TNF-α, tumor necrosis factor alpha; TUNEL, terminal deoxynucleotidyl transferase dUTP nick-end labeling; UATmax, universal apparent maximum temperature; WNT, Wingless-related integration site signaling pathway.
